# Implementation of evidence-based practice for benign paroxysmal positional vertigo: DIZZTINCT– A study protocol for an exploratory stepped-wedge randomized trial

**DOI:** 10.1186/s13063-018-3099-0

**Published:** 2018-12-22

**Authors:** William J. Meurer, Kathryn E. Beck, Brigid Rowell, Devin Brown, Alexander Tsodikov, Angela Fagerlin, Steven A. Telian, Laura Damschroder, Lawrence C. An, Lewis B. Morgenstern, Misty Ujhely, Laura Loudermilk, Sandeep Vijan, Kevin A. Kerber

**Affiliations:** 1 Department of Emergency Medicine, University of Michigan, TC B1-354 1500 E. Medical Center Drive, Ann Arbor, MI 48109 USA; 2 Department of Neurology, University of Michigan, Ann Arbor, MI USA; 3 Stroke Program, University of Michigan, Ann Arbor, MI USA; 4 Institute for Healthcare Policy and Innovation, University of Michigan, Ann Arbor, MI USA; 5 Mathematica Policy Research, Washington, DC, USA; 6 Department of Biostatistics, School of Public Health, University of Michigan, Ann Arbor, MI USA; 7 Department of Population Health Sciences, University of Utah, Salt Lake City, USA; 8 Salt Lake City VA Center for Informatics Decision Enhancement and Surveillance (IDEAS), Salt Lake City, USA; 9 Department of Otolaryngology, University of Michigan, Ann Arbor, MI USA; 10 Implementation Pathways, LLC, Ann Arbor, USA; 11 Department of Internal Medicine, University of Michigan, Ann Arbor, USA; 12 Center for Health Communication and Research, University of Michigan, Ann Arbor, USA; 13 Department of Internal Medicine, Division of General Medicine, University of Michigan, Ann Arbor, USA

**Keywords:** Benign paroxysmal peripheral vertigo, Emergency medicine, Clinical trial, Stepped wedge

## Abstract

**Background:**

Benign paroxysmal positional vertigo (BPPV) is the most common peripheral vestibular disorder, and accounts for 8% of individuals with moderate or severe dizziness. BPPV patients experience substantial inconveniences and disabilities during symptomatic periods. BPPV therapeutic processes – the Dix-Hallpike Test (DHT) and the Canalith Repositioning Maneuver (CRM) – have an evidence base that is at the clinical practice guideline level. The most commonly used CRM is the modified Epley maneuver. The DHT is the gold standard test for BPPV and the CRM is supported by numerous randomized controlled trials and systematic reviews. Despite this, BPPV care processes are underutilized.

**Methods/design:**

This is a stepped-wedge, randomized clinical trial of a multi-faceted educational and care-process-based intervention designed to improve the guideline-concordant care of patients with BPPV presenting to the emergency department (ED) with dizziness. The unit of randomization and target of intervention is the hospital. After an initial observation period, the six hospitals will undergo the intervention in five waves (two closely integrated hospitals will be paired). The order will be randomized. The primary endpoint is measured at the individual patient level, and is the presence of documentation of either the Dix-Hallpike Test or CRM. The secondary endpoints are referral to a health care provider qualified to treat dizziness for CRM and 90-day stroke rates following an ED dizziness visit. Formative evaluations are also performed to monitor and identify potential and actual influences on the progress and effectiveness of the implementation efforts.

**Discussion:**

If this study safely increases documentation of the DHT/CRM, this will be an important step in implementing the use of these evidenced-based processes of care. Positive results will support conducting larger-scale follow-up studies that assess patient outcomes. The data collection also enables evaluation of potential and actual influences on the progress and effectiveness of the implementation efforts.

**Trial registration:**

ClinicalTrials.gov, ID: NCT02809599. The record was first available to the public on 22 June 2016 prior to the enrollment of the first patients in October 2016.

**Electronic supplementary material:**

The online version of this article (10.1186/s13063-018-3099-0) contains supplementary material, which is available to authorized users.

## Background

Benign paroxysmal positional vertigo (BPPV) is the most common peripheral vestibular disorder with a lifetime prevalence of 2.4% [1]. BPPV accounts for 8% of individuals with moderate or severe dizziness [1]. “Benign” is a misnomer in the label of “benign paroxysmal positional vertigo.” BPPV patients experience substantial inconveniences and disabilities during symptomatic periods [1, 2]. Nearly one in four BPPV patients stop driving a car, one in three miss work, and more than three in four seek medical consultation [1].

BPPV processes – the Dix-Hallpike Test (DHT) and the Canalith Repositioning Maneuver (CRM) – have an evidence base that is at the clinical practice guideline level [3, 4]. The most commonly used CRM is the modified Epley maneuver. The DHT is the gold standard test for DHT and the CRM is supported by numerous randomized controlled trials (RCTs), and systematic reviews [5–12].

The problem is that BPPV processes are substantially underutilized. Evidence from our work and others indicates substantial underutilization of the DHT and CRM [1, 13]. Prior epidemiological studies indicate that less than 10% of BPPV patients are treated with the CRM [1]. Our preliminary studies indicate that 78% of patients diagnosed with BPPV in the emergency department (ED) did *not* have the DHT documented and 96.1% did *not* have a CRM documented. The reasons for the underuse of these processes has not been systematically studied and is likely to be complex, involving several constructs including knowledge gaps, clinical inertia, and low provider self-efficacy. The DHT is used to identify BPPV**.**

### Identification of BPPV: the Dix-Hallpike Test (DHT)

The DHT is the gold standard test for BPPV [3, 4]. It is a simple bedside test. A positive test is indicated by up-beating and torsional nystagmus lasting about 10–20 s. Even when physicians use the DHT, there is the possibility that they may not interpret the results correctly [14–16]. Common errors include calling the test positive for symptoms (rather than nystagmus), and making a BPPV diagnosis when there is any pattern of nystagmus observed [17]. Clinicians must be aware that different patterns of positional nystagmus can be triggered by other disorders. For example, patients with vestibular neuritis have horizontal and persistent (not transient) nystagmus that may be most apparent during positional testing. Central disorders can also cause positional nystagmus, typically persistent – not transient – down-beating nystagmus.

### Treatment of BPPV: the Canalith Repositioning Maneuver (CRM)

The CRM is the treatment for BPPV. The CRM is used to move the canaliths from the inferior portion of the involved posterior canal back into the central chamber of the inner ear [3]. In this location, the positional vertigo no longer occurs. The first two steps of the CRM are the same as the DHT. If the DHT is positive on the right side, then there are only three more steps that are used to move the particles out of the canal.

### Relevance and priority for this study

The topic is high impact in terms of the number of patients affected (BPPV lifetime prevalence is 2.4% [1]), efficacy of the CRM [3, 4], and health care efficiencies [1, 13, 18–20]. These factors, and low utilization in the ED setting, justify implementation research. We bring together investigators of multiple disciplines – including emergency medicine (academic and community practice), neurology, otolaryngology (ENT), general medicine, behavioral science, and implementation science – with the goal of helping physicians address a problem they have declared to be a top priority for decision support and which is associated with high frequency of unnecessary testing, such as head computed tomography (CT) scanning and low frequency use of evidence-based practices [1, 13, 18–21]. This project could have a direct positive impact on the effectiveness and efficiency of care for BPPV presentations, and others.

Frontline physicians want support for vertigo. A survey of ED physicians about priorities for the development of clinical decision support (1150 respondents) ranked vertigo as the #1 topic in adult ED presentations [21]. The “lowest-hanging fruit” in the opportunity to achieve meaningful improvements in dizziness presentations is BPPV. BPPV is common, and readily identifiable and treatable at the bedside. No laboratory or imaging studies are needed, and in fact these are explicitly discouraged in guideline statements [4]. ED physicians have strongly advocated for the use of BPPV processes (even stopping an ED-based trial for ethical reasons given the effect size at interim analysis) [5], and our survey (preliminary studies) indicates high demand for BPPV intervention.

### Supporting data

Two evidence-based guidelines support the DHT and the CRM to diagnose and treat BPPV. Evidence-based guidelines supporting the DHT and CRM were published in 2008 by the American Academy of Otolaryngology-Head and Neck Surgery and the American Academy of Neurology [3, 4]. Additional systematic reviews also support the DHT and CRM [6, 7, 12, 22, 23]. The primary RCTs demonstrate the resolution of BPPV symptoms (outcomes measured at 1 day to 4 weeks) in patients treated with the CRM [9–11, 24, 25]. In these studies, 61 to 80% of treated patients had resolution after just one treatment compared with 10 to 48% of untreated patients. These effect sizes translate in to a number-needed-to treat ranging from 1.4 to 3.7, which is among the most substantial effects achievable in clinical medicine. In the study assessing outcome at 24-h, 80% of treated patients were cured versus only 10% of controls [24]. Substantial benefit has also been demonstrated in RCTs from primary care settings [5, 8].

### Aims and objectives

#### Primary objective

The primary hypothesis focuses on BPPV diagnosis is that we will observe different proportions of the primary endpoint (documentation of the DHT or CRM) in ED visits before and after the intervention.

#### Secondary objectives

- Main secondary hypothesis (focuses on BPPV treatment): we will observe different proportions of the secondary endpoint (documentation of the DHT or CRM or outpatient referral for BPPV evaluation to an appropriate provider) in ED visits before and after the intervention. This objective is broader than the primary as it allows referral. The analysis plan will provide the specific number of patients having each of the three components (DHT, CRM, or referral – see the first exploratory objective below)
- Main safety objective – to determine the cumulative incidence of short-term (90-day) stroke within the ED dizziness population aged 45 years and older, both before and after the intervention

#### Exploratory objectives

- Characterize utilization in each individual type of BPPV care process (DHT, CRM, or referral to BPPV provider) across the treatment groups
- Characterize proportions of visits with BPPV process utilization at individual provider (physician, resident, or advanced practice provider) level, both before and after the intervention
- Characterize changes in length of stay in the ED associated with the intervention, both before and after the intervention
- Characterize changes in the utilization of neuro-imaging studies and hospital admission, both before and after the intervention
- Characterize changes in overall costs, both before and after the intervention
- Determine the cumulative incidence of repeat ED dizziness visits, both before and after the intervention
- Determine the proportion of index visit stroke diagnosis in the ED dizziness population in the population aged 45 years and above
- Estimate the proportion of cases receiving documentation consistent with guideline concordant care (composite of the DHT performed in patients without nystagmus or focal neurological deficit; DHT only interpreted as positive with positive documentation of triggered, transient nystagmus)
- Estimate relationship between intensity of intervention contact (time spent on website, attendance at continuing medical education (CME) sessions, hours of academic detailing provided) and change in BPPV diagnostic and treatment endpoints before and after the intervention
- Describe adverse events proximally related to the performance of the DHT or CRM in the ED

## Methods/design

### Design overview

**Fig. 1:**
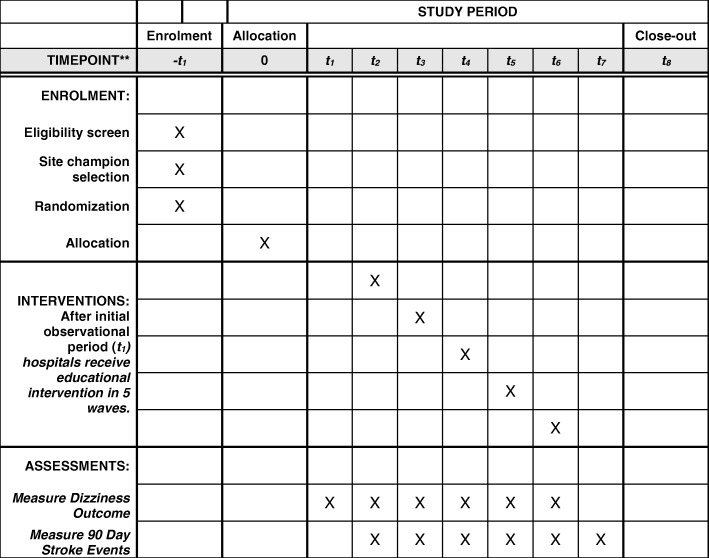
Timing of study events at hospital level

This is a partnered best-practice implementation study, meaning that the local providers will be engaged in the intervention components. The testing design method is a stepped-wedge, randomized clinical trial of a multi-faceted educational and care-process-based intervention designed to improve the guideline-concordant care of patients with BPPV in the ED. The stepped-wedge design has the advantages of being acceptable to stakeholders when the intervention implements evidence-based practice, initiation of the intervention at multiple time points (when a single time point is not logistically feasible), and including contemporaneous controls to evaluate and adjust for secular trends [26]. For this study, the delivery of the intervention to all sites also had the advantage of enabling us to explore variation in implementation fidelity across the mix of sites. The unit of randomization is the hospital. After an initial observation period, the six hospitals will undergo the intervention in five waves (the two closely integrated hospitals will be paired) – see Fig. 1. The order in which each hospital receives the intervention will be randomized. The intervention will be provided as a complete package during the month that each hospital is randomized.

The current design will allow us to estimate the amount of change in the use of guideline-concordant BPPV care processes that is associated with the delivery of the intervention. Each hospital will serve as its own control providing pre and post observations. Since this is stepped-wedge, some hospitals will contribute many more pre-intervention observations and this will allow us to observe for underlying secular trends to ensure that changes observed in care processes are attributable to the interventions.

The focus of the intervention is on health systems and frontline medical providers; however, the observations and outcomes are derived from the medical records of identified cases of dizziness in the included EDs. As patients are not directly intervened upon, and the intervention is to improve guideline-concordant care, we use a waiver of informed consent for the collection of patient-level outcomes from the medical record, along with a waiver of documentation of informed consent for brief phone interviews and Short message service (SMS) and online surveys with patients.

Health care providers involved in the study will be informed of the voluntary nature of this research and will not be required to use any tools or attend any educational sessions provided by this team (see Additional file 1: Medical provider protocol).

### Patient population and setting

The setting for this study is the six hospital-affiliated, EDs in Nueces County, TX, USA. The largest city in Nueces County is Corpus Christi. The community of Corpus Christi was initially identified as an ideal location for these population-based studies because of the geographic isolation from other cities and the focus on representative community practice. These characteristics mean that the majority of acute illness presentations by Nueces County residents will occur within the Nueces County medical facilities and the medical care provided will be more generalizable to other communities than the care that is provided at large, tertiary-care referral centers. Given our extensive prior work in Nueces County, we have also developed long-standing relationships with providers, administrators, and other persons in Nueces County. The University of Michigan has a field office in Corpus Christi with approximately 10–15 full-time employees.

### Eligibility and enrollment

The dizziness visits are the unit of analysis for the primary measure of use of BPPV processes by the health care providers.

#### Inclusion criteria for dizziness visit medical record review and data abstraction

- Aged 18 years or older
- ED patient seen at one of six full-service, non-freestanding EDs in Nueces County, TX, USA
- Principal dizziness case: the triage reason for visit is a dizziness symptom *or* a dizziness symptom is one of the first three listed complaints in physician medical record *or* a dizziness diagnosis (e.g., dizziness or vertigo not otherwise specified (NOS), BPPV, vestibular neuritis) is recorded as one of the first three final ED diagnoses

#### Exclusion criteria

- Prisoners
- Cognitively impaired adult (defined for study purposes as notation of mental retardation or similar diagnosis within the chart)

### Study procedures

Hospital inclusion **–** Data collection will commence in the pre-intervention period and at that point, the hospitals will be engaged in research.

#### Identification of cases

Cases of dizziness presentations will be captured by active and passive surveillance. Study abstractors will be blinded to randomization periods and groups. For active surveillance, ED logs will be screened for dizziness terms (e.g., dizziness, vertigo, imbalance, spinning, lightheadedness, nausea, common misspellings or similar terms) as the patients’ reasons for visiting the ED. Passive surveillance will be performed by screening an *International Classification of Diseases, versions 9/10* (ICD-9/10) hospital database for dizziness and vestibular ICD-9/10 codes. We do not limit the population to patients receiving a BPPV diagnosis for the following reasons: the likelihood of diagnostic misclassification [17, 20], the high frequency of cases receiving symptom only diagnoses (see preliminary results), and diagnostic bias introduced by the intervention during the post-intervention phase (e.g., providers may be more likely to use BPPV diagnoses after the intervention)*.*

#### Screening and enrollment

All cases meeting the inclusion criteria and the definition of principal dizziness visit will undergo data abstraction and will be included in the overall trial database. We will collect monthly counts of the number of charts with any screening dizziness terms at each site.

#### Consent/assent procedures – patient-level data collection

Patients will be receiving routine clinical care at the local EDs and we have sought a waiver for review and collection of data from the medical charts. For follow-up telephone surveys of patients in the ED, we will use a structured script to provide information about the study and get informed consent to ask brief questions about the performance of BPPV care processes in the ED. For the SMS and online surveys, patients will be asked to opt in if they are willing to complete the study questions.

#### Intervention group assignment

Patients will be assigned to either the intervention or control group based on the time and hospital they present to for their dizziness care. If the hospital has received the intervention and is in the post-intervention period – they will be in the intervention group; otherwise, they will be considered control patients (pre-intervention). The hospitals will receive the intervention at a randomly assigned time point.

### Study interventions

The overall educational intervention is delivered at the hospital level. Table 1 provides an overview of the hospital-level interventions that occur as part of the trial. Attendance by medical providers will be tracked to determine utilization.

**Table 1:** Multi-faceted intervention overview

Implementation strategy components	Component description
1. Local champions	Local champions (ED providers) will be recruited and trained in BPPV testing and diagnosis. Each will participate in the CME session, follow-up sustainability session, and commit to help during routine care
2. Educational sessions. Interactive and hands-on sessions	Sessions will review BPPV mechanisms and evidence, utilize videos, include hands-on demonstration, and address barriers from aim 1
3. Decision aid. Multi-media web-based decision aid application	Includes high-yield text and videos on the BPPV processes and characteristic exam findings. Audit and Feedback – split out as its own row. Will also include individual- and group-level feedback on dizziness care process delivery over time
4. Referral resource	Readily available list of outpatient, experienced, BPPV providers accepting referrals
5. Follow-up educational sessions	Sessions, led by local champions, to facilitate adoption, implementation, and sustainability
6. Partnered development and other resources	Other resources, identified by and developed by the local medical providers may be provided

*BPPV* benign paroxysmal positional vertigo, *CME* continuing medical education, *ED* emergency department

#### Champion development

Local ED physician champions will be recruited for each hospital system. These champions will serve as the point person of contact for the local ED physician groups, will receive more focused training on BPPV prior to the CME sessions, will be a key participant (instructor) at the CME session, will lead follow-up educational sessions, and be available for questions as needed.

#### Interactive high-yield, hands-on education session (CME session)

An evidence-based educational session will be developed to be presented by Drs. Kerber, Meurer, and local champions to the Corpus Christi ED physicians, physicians assistants, and residents and will be used to introduce the decision aid. Common barriers will be addressed along with suggestions for overcoming barriers. For example, we anticipate that one barrier in the ED will be small examination gurneys. Throughout the session, videos will be used to enhance learning. Videos will demonstrate the DHT and CRM, the characteristic BPPV nystagmus, and other eye movements that can be misinterpreted as BPPV nystagmus (e.g., eyelid blinks, voluntary movements, and nystagmus patterns of vestibular neuritis, or a central lesion). Descriptions of factors that could lead to misclassification of BPPV cases based on the DHT will be provided, such as extremely slow movements or basing the test interpretation on symptoms rather than the characteristic pattern of nystagmus. A specific part of the session will focus on risks of the BPPV processes and dangerous mimickers of BPPV. It will be made clear that the processes should be considered contraindicated in patients with known or suspected cervical spine instability until spinal stability has been ascertained. Interactive techniques will be incorporated to maximize the probability of outcome success by adhering to principles of adult education: delivering content in a learner-centered, active format, relative to the learner’s needs, which is simultaneously engaging and reinforcing [26]. The sessions will target increasing self-efficacy (perception of one’s abilities) and outcome expectancy (belief that a behavior will lead to the desired outcome). High outcome expectancy is associated with an increased likelihood of performing a behavior [27]. Hands-on training for the DHT and CRM will also be developed as part of the presentation. Physicians will pair up at tables and perform the DHT and CRM under instructor guidance (i.e., investigators and champions). Models of the semicircular canals will be used to facilitate understanding the basis for the processes. CME credit and a modest incentive US$50 will be offered to encourage medical provider attendance. Individual attendance will be recorded. To promote the widest exposure of the educational sessions, the sessions will also be video recorded and made available for post-intervention physicians and medical providers to review on their own time. In addition, individual or small group sessions will be offered for providers not able to attend the primary sessions. To improve later presentations, the project team will review recordings of CME sessions.

#### Web-based, multi-media, real-time decision aid

We have developed a web-based application in accordance with the findings from physician interviews and environment barrier assessments (http://www.dizztinct.com). This aid is a tool providers will be able to efficiently use at the point of care. Extensive collaborations with our behavioral scientists and technology developers informed the content and structure of this aid. We plan for providers to be able to use the aid in less than 10 min, though it will also include additional resources and information so that more details are available for interested providers. The aid uses videos and includes brief, high-yield narration, video instructions on the DHT and CRM, and video demonstrations of positive and negative test results. The videos demonstrate dangerous signs of central nervous system disorders. The aid also highlights potential risks of the processes and BPPV mimickers. Additionally, it states that known or suspected cervical spine instability is a contraindication until spinal stability has been ascertained. The aid will be password protected to each individual’s identity.

The aid will also contain tailored data on the individual’s performance on dizziness care processes. The proportion of eligible dizziness cases receiving the DHT (primary BPPV diagnostic endpoint) and the CRM (secondary BPPV therapeutic endpoint), will be plotted over time. This will be graphically summarized to demonstrate the performance of all health care providers (anonymized to user) at the site over time, and the individual data point corresponding to the user will be noted in a monthly email and within the website. The aid will also be available in a mobile version for smartphones and tablets.

#### Referral resource

Because our main goal is to get the right treatment to the right patient within a reasonable time frame, we will also establish a list of providers in the community who evaluate and treat BPPV. The goal of this system is to provide ED physicians with a more informed route of referral to appropriate community providers for BPPV treatment. Many providers who see patients with BPPV or probable BPPV would like to make a specific referral to another provider for evaluation, treatment, or subsequent assessment. Our previous survey work of community ED providers found this to be a popular option. To establish this resource, providers in the community who evaluate and treat BPPV will be identified, covering a variety of insurance/payment options. Physical therapists, particularly those with training in vestibular therapy, are likely to be an important resource. We have already been in contact with PTs in the community in this regard. A system will be established to track use of referrals. In general, we will use the existing hospital methodology for referrals; however, we may be able to augment this with a list of local providers who are willing and able to quickly see and treat these patients.

#### Follow-up educational sessions

Follow-up maintenance sessions will also be developed for the time period after the first CME session. These sessions will be used to facilitate adoption, implementation, and sustainability. The format of follow-up sessions will be case-based. Providers will be encouraged to express successes and failures. Feedback on BPPV processes utilization will be presented. These sessions may be led by the designated local champion, an interested local medical provider, or the study team. These sessions may be in person or by telephone or web conference.

#### Partnered adaptations of other resources identified by local medical providers

This is a partnered implementation project, and based on our engagement with the providers during the project we expect to learn even more about provider preferences and needs based on provider feedback. The intervention components can be adjusted/adapted based on provider feedback and requests, consistent with the approved protocol for this research. For example, it is possible that providers may request additional resources or patient-specific materials. If they make such a request, we will help them in finding/developing/implementing such other resources. We, as the researchers, will not generate such materials ourselves without proper regulatory approval.

#### Dizziness Checklist (introduced in protocol version 2.0)

In the course of engaging with providers, they requested an additional website/app component to prospectively collect information related to safety in discharging ED dizziness patients home. We worked with providers to develop a list of items and a data collection form. The list of items includes the BPPV-specific items and additional items that largely relate to the possibility that the patient might have a stroke as the cause or is high risk for stroke in the short term. Both of these components fit well with our overall intervention which emphasizes both the features of BPPV and also findings that suggest an alternative cause such as stroke. The addition of this component could serve to: (1) increase exposure to the BPPV related items (an additional resource providers may seek out), (2) enhance data capture in a subsample of visits, and (3) build engagement/collaboration with local providers. The new prospective data collection could enhance implementation fidelity measures because this prospective provider-entered data enables an assessment of the consistency of medical record documentation of BPPV assessments. In addition, the new prospective data regarding safety items should enhance secondary analysis regarding the 90-day stroke rate by informing factors that predict stroke (in the subsample with data collected).

In the form, we separate the BPPV items from the safety items to enforce that the BPPV items are established clinical guideline items but that the safety items are not. The form also explicitly states that the safety items are for data collection purposes only. There is no current society clinical guideline regarding safety in discharge of ED dizziness patients. To develop these items, we reviewed the medical literature regarding factors previously shown to predict stroke. The items were then created/edited by our team of investigators (neurologists, emergency medicine, ENT, general medicine) and local ED providers. If the analysis of these safety items indicates they are accurate in discriminating stroke, then future studies (requiring separate Institutional Review Board (IRB) application) may be done to test the effect of the list on outcomes in dizziness ED cases (e.g., subsequent stroke event, length of stay in the ED, test utilization).

The form will be available on the website/app. Providers can voluntarily complete the form at the point of care. Data can be entered electronically, or paper forms can be printed, completed, and inserted into a lock-box in the ED.

#### Randomization

The overall study period will be approximately 18 months. The first 4 months is the pre-intervention period. After this, the sites will receive the intervention in five waves occurring approximately every 2 months. The randomization sequence was generated using a specific R program written by WJM with a specific random number seed (to allow for reproducibility). (Two closely integrated hospitals which have almost complete overlap in providers will receive the intervention at the same time.) Finally, there will be approximately 4 months where all sites will be in the post-intervention period. Therefore, each site will have distinct pre- and post-intervention times.

#### Concomitant interventions

The community will be closely monitored for interventions that may change the underlying diagnosis or treatment of BPPV (i.e., a health insurer or malpractice-insurer-based intervention on the care of dizziness in the ED.) The primary analysis will remain unchanged, but secondary analyses will be conducted to estimate the impact of this change within hospitals that have and have not received the intervention by the time of the event.

#### Adverse experiences

The definitions of adverse events (AEs), and serious adverse events (SAEs) are available in the protocol as Additional files 1, 2, 3, and 4.

### Statistical considerations

#### Endpoint

The primary endpoint (BPPV diagnosis) is measured at the individual patient level, and is the presence of documentation of either the DHT or CRM (since the first step of the CRM is the diagnostic maneuver).

Secondary endpoints (BPPV treatment) are composite (any) of following three items: (1) Documentation of DHT alone in the ED, (2) Documentation of CRM during ED visit, (3) Outpatient referral to a BPPV provider (ENT surgeon, neurologist, physical therapist).

Safety secondary endpoint: 90-day cumulative incidence of stroke in patients aged 45 years and above following initial ED discharge home visit for dizziness. Validated stroke event will be obtained by data linkage with an on-going stroke surveillance study in the same medical systems: The Brain Attack Surveillance In Corpus Christi (BASIC) project [27].

Additional exploratory endpoints: each of the individual components of the composite secondary endpoint (DHT, CRM, or referral), stroke at index ED dizziness visit, ED length of stay in hours, neuro-imaging utilization, and inpatient hospitalization utilization.

Validity of primary endpoint: the primary endpoint has been rigorously evaluated in our preliminary work [28]. The documentation of performance of BPPV care processes is rare, and, in discussion with physicians in emergency medicine practice, the rarity of documentation is well reflected in clinical practice: BPPV care processes are rarely used and rarely documented. In addition, a second reviewer who was blinded to treatment group assignment (pre versus post intervention based on time within site) reviewed each chart to determine the presence or absence of the primary endpoint.

#### Sample size and accrual

The trial will start with an initial no intervention period of approximately 4 months followed by randomized staggered intervention with a new hospital entering approximately every 2 months, finalized by approximately four post-intervention months will result in the approximately balanced number of 867 visits occurring without intervention and 933 visits occurring under (post) intervention. We chose this timing to balance the amount of overall resources (grant funding) we had for data collection with having adequate separation between each intervention and having a sufficient post-intervention period to observe response to intervention. This calculation assumes the average anticipated total patient visit rate of 100 patients per month. Based on our pilot studies and the literature we expect the DHT or CRM procedure to be done in 5% patients before the intervention. With the expected number of visits calculated above, we will be able to detect the increased DHT or CRM rate of 9% and above with 90% power by a two-sided test at the significance level of 5%. The reserves of power will be used to provide more power to fine-tune the multivariate mixed-regression models and associated secondary analyses.

Patients: each month, approximately 100 patients with dizziness seek care at the six-hospital associated-EDs in Nueces County, TX, USA. Over an 18-month period, we anticipate a total sample size of approximately 1800. Approximately half of the 1800 patient visits will occur prior to the intervention in the overall study. As dizziness volume can vary widely both within and across EDs we have provided maximum sample sizes for each site that are 200% of our initial estimates. After evaluating initial accrual, a higher number of ED visits for dizziness met our inclusion criteria, and as such we are now providing increased maximum sample sizes. Our revised total expected enrollments will be 6800 with a revised maximum of 10,800. We believe the higher-than-normal number of visits likely relates to a lower threshold for inclusion in the current study than in the study used for preliminary estimates and an increase in dizziness presentations to the ED now compared with time of preliminary estimates. In addition, a temporary closing of one of the EDs is resulting in higher volumes in the others EDs.

#### Data monitoring

The trial does not employ formal efficacy or futility stopping rules. As a cluster-delivered educational intervention to several hospitals, formal stopping boundaries for efficacy or futility are not appropriate.

The Independent Medical Monitor (IMM) will review primary endpoint data by group. The principal investigators (PIs) will review overall primary outcome data and will remain blinded to performance by group. In addition, the main safety endpoint (90-day stroke rate) will be included for reporting to the investigators and the IMM; it will be further classified as immediate diagnosis (at time of index ED primary dizziness visit) or delayed diagnosis (at some point during following 90 days). A brief narrative will be provided with each stroke diagnosis, based on the linked record from the BASIC study (i.e., 50-year-old woman with a middle cerebral artery stroke 85 days after ED primary dizziness visit). A summary of SAEs and AEs meeting the DIZZTINCT definition for inclusion will also be routinely monitored by the PIs and the IMM. Data quality will be assessed by routine monitoring by the study project monitor, along with review of the interview data that assesses patients directly for the performance of BPPV care processes.

#### Data analyses

The intervention is delivered to hospitals. Intervention is a binary variable with two levels, pre-intervention (no intervention), post-intervention (under intervention).

The primary analysis will use binary logistic regression and will include covariates for hospital, month (to handle secular trends), and intervention (see below). Provider (attending physician) will be included as a random effect (intercept). For a set of new patient visits, the binary random variable DHT/CRM “yes/no” will serve as the primary response. Patient visits will be supplied with patient-, hospital- and provider-level covariates as well as the calendar time variable modeling the secular trend, and the intervention yes/no variable measuring whether the visit occurs under intervention or not. To take hospital- and provider-specific unmeasured features into account, hospital categorical variables will be included in the analysis and we will include random intercept for provider. Due to the fact that the number of hospitals and providers is much smaller than the number of patient visits, adjusting for hospital and provider effects by way of categorical variables will not lead to bias. Secondary analyses will explore alternative approaches using random effects (Gaussian) models. A two-sided model-based test for the intervention variable will be used to test the primary hypothesis at the significance level of 5%. In addition, we will report the observed proportions of the primary and secondary endpoints and the differences in proportions stratified by treatment versus control. We do not plan to adjust for repeated visits by a patient, as our primary endpoint was measuring provider actions (and repeated ED visits by an individual patient over an 18-month period are likely to have different symptoms and disease processes.) We will create graphical plots of changes in the primary, secondary, and selected exploratory endpoints over time.

The secondary analysis (safety) will numerically summarize the 90-day stroke rate – cumulatively and stratified for stroke diagnosed on the index dizziness visits and for post-index visit strokes (delayed diagnosis) in patients seen at EDs with and without the intervention. This is anticipated to be very rare. The intervention does not target improving stroke diagnosis. However, evaluating both the index visit stroke diagnosis rate and the delayed diagnosis rate should allow for determination of major changes. We anticipate the index visit stroke diagnosis rate to be approximately 2–3% and the delayed diagnosis rate to be approximately 0.6% [29, 30]. Additional details of the planned analysis are included with the draft statistical analysis plan (which will be finalized prior to the end of recruitment) as Additional files 1, 2, 3, and 4.

#### Cost analysis

Cost analysis will be performed including inputs of tests, medications, procedures, consultations, and level of visit. Additional costs will also be gathered relating to hospital admission and subsequent visits to the ED. Unit costs will be derived from nationally representative sources (e.g., Medicare fee schedule). Costs often have a skewed distribution and thus will be transformed (typically a log transformation) to achieve a more normal distribution. The costs in patients treated by pre-intervention providers will be compared to costs in patients treated by post-intervention providers using the *t* test and also a generalized linear model (GLM). General concepts for modeling costs will be followed, including adjusting for secular trends.

#### Formative evaluations (FE)

We will conduct implementation- and progress-focused formative evaluations during the course of implementation to monitor and identify potential and actual influences on the progress and effectiveness of the implementation efforts [31]. We will monitor implementation fidelity through attendance at initial and follow-up educational sessions, the use of the web-based decision aid, and referral networks. Feedback will be solicited and recorded at the initial and follow-up education sessions, and the study team will determine the adjustments/adaptations in the strategy based on this feedback. At the conclusion of the implementation period, we will conduct semi-structured interviews with key stakeholders (e.g., providers, local champions, project manager, referral network members). The data will be analyzed qualitatively using a grounded theory approach intended to inductively find themes that could inform future intervention design or implementation. The knowledge gained from FE will inform needed adjustments, unanticipated consequences, issues, and resolutions for future implementations.

#### Dissemination

The study team will present the results to the providers in the community. In addition, the primary analysis will be presented in a main paper. Data will be available to interested scientists if appropriate data use agreements are executed. Given that we have not collected persistent contact information for the patients who contributed data, we do not have plans to directly contact them. Despite this, we will plan for press release(s) within the community to share the results.

## Discussion

This implementation research study uses a stepped-wedge, randomized clinical trial design to assess the impact a multi-faceted educational and care-process-based intervention on the documentation of the DHT and CRM in ED visits for dizziness. We plan to use the results of this study to first determine whether it would be worthwhile to proceed to a larger-scale study designed to change physician practice within multiple health systems. Key strengths of our approach is our use of a community-based cohort of patients and health care providers, and the addition of our infrastructure to an existing stroke surveillance study – allowing us to more efficiently ensure that we did not increase the proportion of patients who had missed strokes.

### Limitations

The trial as designed, has some important limitations. First, we only included six hospitals within a single geographic community in the United States – and, as such, these findings may not be generalizable to other settings. We hope that what we learn from this exploratory phase trial will help us design interventions that can impact a more diverse set of EDs. Second, despite the large number of dizziness patients, we anticipate that the number of outcome events (DHT or CRM) will be relatively small, so larger studies will be needed to more precisely understand the effects of our intervention. Third, we did not allow for a third period surrounding the time of the intervention at each site. Given that the effects of the intervention are likely to be delayed, this means that we will rely on observation of temporal trends within each site to better understand the timing and durability of practice change if the intervention is successful. Some aspects, such as the checklist requested by the community physicians in this study, will not have been in use long enough to be considered validated. As such, future studies may involve prospective validation of some of the study elements. In addition, we will need to use what we have learned in this study to design processes to evaluate effects on patient outcomes. Our study did not evaluate the effects of our intervention on care processes for patients without dizziness and this may be an interesting area for future study. A major assumption of our preliminary study is that by encouraging the use of the inexpensive, and evidence-based DHTs and CRMs, we will translate prior, observed, improved patient outcomes into the emergency setting. We will need to confirm the validity of this assumption in future trials.

## Trial status

Recruitment began in October of 2016. This manuscript was initially submitted in March 2018. We anticipate accrual will continue to April 2018. We will evaluate for stroke in accrued patients for 90 days following the end of accrual. No protocol or statistical analysis plan changes occurred between the submission of this paper and the completion of data collection and analysis, with the exception of the administrative adding or deletion of personnel.

## Additional files

Additional file 1: Medical provider protocol. (PDF 250 kb)

Additional file 2: Patient-level data collection protocol. (PDF 351 kb)

Additional file 3: Preliminary statistical analysis plan. (PDF 214 kb)

Additional file 4: Standard Protocol Items: Recommendations for Interventional Trials (SPIRIT) 2013 Checklist: recommended items to address in a clinical trial protocol and related documents*. (DOC 123 kb)

## Abbreviations

AE: Adverse event
BASIC: Brain Attack Surveillance in Corpus Christi
BPPV: Benign paroxysmal positional vertigo
CME: Continuing medical education
CRM: Canalith Repositioning Maneuver
DHT: Dix-Hallpike Test
ED: Emergency department
ENT: Ear, nose, and throat (otolaryngology)
GLM: Generalized linear model
ICD: *International classification of diseases*
IMM: Independent Medical Monitor
IRB: Institutional Review Board
NOS: Not otherwise specified
SAE: Serious adverse event
SMS: Short message
