# Supplementary material for: Implementation of evidence-based practice for benign paroxysmal positional vertigo: DIZZTINCT– A study protocol for an exploratory stepped-wedge randomized trial
Source: Trials. 2018 Dec 22;19:697. doi: 10.1186/s13063-018-3099-0 (PMC6303863; doi:10.1186/s13063-018-3099-0)
Supplement: Supplementary file 2 — Patient-level data collection protocol. (PDF 351 kb) [file 13063_2018_3099_MOESM2_ESM.pdf]

Partnered Implementation of Evidence Based Best Care Practice for Benign Paroxysmal Positional Vertigo: A Stepped Wedge, Randomized Controlled Clinical Trial.
[Dizziness Visit Level Component]

Study Investigators

University of Michigan: Principal Investigators: Kevin Kerber, MD, MS; William Meurer, MD, MS.

Supported by:

The National Institute on Deafness and Other Communication Disorders (NIDCD)

R01 DC012760

Study Intervention Provided by:

The Regents of The University of Michigan

Sponsor of IND (IDE):

Not applicable

(Any modification to the protocol should be annotated on the coversheet or in an appendix. The annotation should note the exact words that are changed, the location in the protocol, the date the modification was approved by the Executive Committee, and the date it became effective.)

Version 2.0 14 Dec 2017

- **Study Investigators 1**

Supported by: **1**

The National Institute on Deafness and Other Communication Disorders (NIDCD) **1**

Study Intervention Provided by: **1**

The Regents of The University of Michigan **1**

Version 1.1 15 March 2016 ..... **1**

SYNOPSIS **4**

1 STUDY OBJECTIVES **7**

1.1 Primary Objective *7*

1.2 Secondary Objectives *7*

1.3 Exploratory Objectives *7*

2 Background **8**

2.1 Rationale *8*

2.2 Supporting Data *10*

3 STUDY DESIGN **11**

4 SELECTION AND ENROLLMENT OF SUBJECTS **11**

4.1 Inclusion Criteria for Dizziness Visit Medical Record Review and Data Abstraction *11*

4.2 Exclusion Criteria *12*

4.3 Study Enrollment Procedures *12*

5 STUDY INTERVENTIONS **13**

5.1 Interventions, Administration, and Duration *13*

5.2 Randomization *17*

5.3 Concomitant Interventions *17*

6 CLINICAL AND LABORATORY EVALUATIONS **17**

6.1 Timing of Evaluations – Patient level *17*

6.2 Timing of Evaluations – Hospital Level *18*

7 MANAGEMENT of adverse experiences **19**

7.1 Definition of Adverse Event and Serious Adverse Event and Scope of AE reporting *19*

7.2 Unexpected (Unanticipated) Adverse Event *19*

This work is licensed under a Creative Commons Attribution-NonCommercial-NoDerivatives 4.0 International License.

7.3 Severity of an Adverse Event *19*
7.4 Classification of an Adverse Event *20*
7.5 Relationship to Study Treatment *20*
7.6 Adverse Event Recording and Reporting *21*
7.7 Expected / Anticipated Adverse Events Associated With Dizziness Care Processes *21*

8 STATISTICAL CONSIDERATIONS 22
8.1 General Design Issues *22*
8.2 Endpoint *22*
8.3 Sample Size and Accrual *23*
8.4 Data Monitoring *24*
8.5 Data Analyses *25*

9 DATA COLLECTION, SITE MONITORING, AND ADVERSE EXPERIENCE REPORTING 25
9.1 Records to Be Kept *25*
9.2 Role of Data Management *26*
9.3 Quality Assurance *26*
9.4 Adverse Experience Reporting *26*

10 HUMAN SUBJECTS 26
10.1 Risks *26*
10.2 Adequacy of Protection Against Risk *28*
10.3 Institutional Review Board (IRB) Review and Informed Consent *29*
10.4 Subject Confidentiality / Data Retention *29*
10.5 Study Modification/Discontinuation *29*

11 IndePEndent Medical Monitor PLAN 29

12 PUBLICATION OF RESEARCH FINDINGS 30

13 REFERENCES 30

Appendix A – Consent / Survey Script 32

Appendix B Independent Medical Monitor Plan 35

This work is licensed under a Creative Commons Attribution-NonCommercial-NoDerivatives 4.0 International License.

Listing of changes

Version 1.2:

Changed from grouping two smallest hospitals for intervention to grouping two closely integrated hospitals (See changes in synopsis and sections 3, 5.2 and 5.3).

Revised maximum sample sizes at each site based on higher than expected accrual rates (See changes in synopsis and section 8.3).

Revised exclusion criteria to reflect exclusion of prisoners and adults with severe cognitive impairment (See changes in section 4.2.2).

Version 2.0:
Add provider checklist (See changes in section 5.1.6).

Added procedures for SMS and online surveys and updated Appendix A (See changes in sections 3, 4.3.4, 6.1.2, 9.1, 10.1 and 10.2).

This work is licensed under a Creative Commons Attribution-NonCommercial-NoDerivatives 4.0 International License.

SYNOPSIS

Study Title:

Partnered Implementation of Evidence Based Best Care Practice for Benign Paroxysmal Positional Vertigo: A Stepped Wedge, Randomized Controlled Clinical Trial.

Objectives

Using a partnered process, the primary objective is to evaluate, in a community ED setting, the effect of the multi-faceted, theory-based educational intervention and implementation strategy on the use of the Dix-Hallpike test and Canalith Repositioning Maneuver in ED dizziness visits. The secondary objectives are to evaluate safety, variation in BPPV process utilization at the provider level, care processes (ED length of stay, neuroimaging, inpatient hospitalizations), and health care costs.

Design and Outcomes

This is a stepped-wedge, randomized clinical trial of a multi-faceted educational and care-process based intervention designed to improve the guideline-concordant care of patients with BPPV in the emergency department. The unit of randomization and target of intervention is the hospital. After an initial observation period, the six hospitals will undergo the intervention in five waves (two closely integrated hospitals will be paired). The order will be randomized. This is a partnered research project with local physicians engaging in best practice implementation.

The primary endpoint is measured at the individual patient level, and is the presence of documentation of either the Dix-Hallpike test or the Epley Maneuver (which contains the Dix-Hallpike as the initial movement.) The secondary endpoints are referral to a health-care provider qualified to treat dizziness for the Epley or CRM and 90-day stroke following an ED dizziness visit (safety). The main exploratory endpoints are cost of care, ED length of stay, neuroimaging utilization, and inpatient hospitalization utilization, and index visit.

The primary hypothesis is that patients seen at hospitals that have received the intervention will have a higher likelihood of receiving a BPPV care process (primary endpoint performance of DHT or CRM in the ED) compared to patients seen at hospitals that have not received the intervention.

Interventions and Duration

The intervention includes six main components: the recruitment and training of local champions who will serve as colleague experts in the community; interactive hands-on educational sessions that address BPPV mechanisms, evidence and practice; a high-yield decision-aid web application

This work is licensed under a Creative Commons Attribution-NonCommercial-NoDerivatives 4.0 International License.

for self study and point of care use; development of a referral network for follow up care; follow-up educational sessions to review successes and barriers amongst the intervention group; and other resources developed locally in partnership with the community medical providers.

Sample Size and Population

Each month, approximately 100 patients with dizziness seek care at the six hospital associated-emergency departments in Nueces County, Texas. Over an 18-month period we anticipate a total sample size of approximately 1,800. From within the 18-months, there will be an initial 4-month observation period, then each of the five hospital groups will receive the intervention in 2-month intervals (10-months) with a final 4-month post-intervention observation period. Approximately half of the 1,800 patient visits will occur prior to the interventions in the overall study. Interim evaluation of accrual, suggests that the total number of dizziness visits in the 18-month period will actually be around 6,800.

This work is licensed under a Creative Commons Attribution-NonCommercial-NoDerivatives 4.0 International License.

1 STUDY OBJECTIVES

1.1 Primary Objective

The primary hypothesis is that patient visits occurring at hospitals that have received the intervention will have a significantly different rate of having the primary endpoint (documentation of the DHT or CRM) associated with the ED care compared to patient visits occurring at hospitals that have not received the intervention.

1.2 Secondary Objectives

● Main secondary objective: We hypothesize that patient visits occurring at hospitals that have received the intervention will have a significantly different rate of having any BPPV care process endpoint (documentation of the DHT or CRM or outpatient referral for BPPV evaluation to an appropriate provider) associated with the ED care compared to patient visits occurring at hospitals that have not received the intervention.

● Main safety objective – to determine the cumulative incidence of short-term (90 day) stroke within the ED dizziness population aged 45 and older, both before and after the intervention.

1.3 Exploratory Objectives

● Characterize utilization in each individual type of BPPV care process (Dix Hallpike, CRM, or referral to BPPV provider) across the treatment groups.
●Characterize variation in BPPV process utilization at provider level, both before and after the intervention.
● Characterize changes in length of stay in the ED associated with the intervention, both before and after the intervention.
● Characterize changes in the utilization of neuro-imaging studies and hospital admission, both before and after the intervention.
● Characterize changes in overall costs, both before and after the intervention.
● Determine the cumulative incidence of repeat ED dizziness visits, both before and after the intervention
● Determine the proportion of index visit stroke diagnosis in the ED dizziness population in the population aged 45 and above.
● Estimate the proportion of cases receiving documentation consistent with guideline concordant care (Composite of Dix-Hallpike test performed in patients without nystagmus or focal neurological deficit; Dix-Hallpike only interpreted as positive with positive documentation of triggered, transient nystagmus);

This work is licensed under a Creative Commons Attribution-NonCommercial-NoDerivatives 4.0 International License.

- Estimate relationship between intensity of intervention contact (time spent on website, attendance at CME sessions, hours of academic detailing provided) and change in BPPV diagnostic and treatment endpoints before and after the intervention.

2 BACKGROUND

Please note: the educational intervention and the collection of data from health care providers is covered in a separate IRB application. This application covers the overall experimental design and the collection of data from patients.

2.1 Rationale

Benign paroxysmal position vertigo (BPPV) is the most common peripheral vestibular disorder with a lifetime prevalence of 2.4%.[1] BPPV accounts for 8% of individuals with moderate or severe dizziness.[1] "Benign" is a misnomer in the label of "Benign Paroxysmal Positional Vertigo". BPPV patients experience substantial inconveniences and disabilities during symptomatic periods.[1,2] Nearly 1 in 4 BPPV patients stop driving a car, 1 in 3 miss work, and more than 3 in 4 seek medical consultation.[1]

BPPV processes – the Dix-Hallpike Test (DHT) and the Canalith Repositioning Maneuver (CRM) – have an evidence base that is at the clinical practice guideline level (See Section 2.2).[3,4] The DHT is the gold standard test for DHT and the CRM is supported by numerous randomized controlled trials, and systematic reviews.[5-12]

The problem is that BPPV processes are substantially underutilized. Evidence from our work and others indicates substantial underutilization of the DHT and CRM.[1,13] Prior epidemiological studies indicate that less than 10% of BPPV patients are treated with the CRM.[1] Our preliminary studies indicate that 78% of patients diagnosed with BPPV in the emergency department (ED) did *not* have the DHT and 96.1% did *not* have a CRM. The reasons for the underuse of these processes has not been systematically studied and is likely to be complex, involving several constructs including knowledge gaps, clinical inertia, and low provider self-efficacy. **The Dix-Hallpike test (DHT) is used to identify BPPV.**

2.1.1 Identification of BPPV: The Dix-Hallpike Test (DHT)

The DHT is the gold standard test for BPPV.[3,4] It is a simple bedside test. A positive test is indicated by up-beating and torsional nystagmus lasting about 10-20 seconds. Even when physicians use the DHT, there is the possibility that they may not interpret the results correctly.[14-16] Common errors include calling the test positive for symptoms (rather than nystagmus), and making a BPPV diagnosis when there is any pattern of nystagmus observed.[17] Clinicians must be aware that different patterns of positional nystagmus can be triggered by other disorders. For example, patients with vestibular neuritis have horizontal and persistent (not transient) nystagmus that may become most apparent during positional testing. Central disorders can also cause positional nystagmus, typically persistent – not transient -- down-beating nystagmus.

This work is licensed under a Creative Commons Attribution-NonCommercial-NoDerivatives 4.0 International License.

2.1.2 Treatment of BPPV: The Canalith Repositioning Maneuver (CRM)

The Canalith Re-positioning Maneuver (CRM) is the treatment for BPPV. The CRM is used to move the canaliths from the inferior portion of the involved posterior canal back into the central chamber of the inner ear.[3] In this location, the positional vertigo no longer occurs. The first two steps of the CRM are the same as the DHT. If the DHT is positive on the right side, then there are only three more steps that are used to move the particles out of the canal.

2.1.3 Patient population

The setting for this study is the six, hospital affiliated, emergency departments in Nueces County, Texas. The largest city in Nueces County is Corpus Christi. We have extensive experience performing research regarding both dizziness and stroke visits to the Corpus Christi emergency rooms from a prior National Institutes of Health K23 award (PI, Kevin Kerber) and the on-going Brain Attack Surveillance in Corpus Christi (BASIC) project (Multiple PIs, Lewis Morgenstern, Lynda Lisabeth). The community of Corpus Christi was initially identified as an ideal location for these population-based studies because of the geographic isolation from other cities and the focus on “real world” practice. These characteristics mean that the majority of acute illness presentations by Nueces County residents will occur within the Nueces County medical facilities and the medical care provided will be more generalizable to other communities than the care that is provided at large tertiary care referral centers. Given our extensive prior work in Nueces County, we have also developed long-standing relationships with providers, administrators, and other persons in Nueces County. The University of Michigan has a field office in Corpus Christi with approximately 10-15 full time employees.

Dizziness surveillance is used to identify visits for dizziness, vertigo, or imbalance to any of the six hospital affiliated EDs in Corpus Christi. Methods previously developed for dizziness surveillance in this community will be used.[18] Data are abstracted from source documents. Cases are classified as dizziness presentations when triage reason for visit is dizziness or the physician documentation form includes dizziness as one of the top three complaints in the physician note or dizziness final diagnosis (i.e., “dizziness”, “vertigo”, or a peripheral vestibular disorder) is rendered by the treating physician.

2.1.4 Relevance and priority for this study

The topic is high impact in terms of the number of patients affected (BPPV lifetime prevalence is 2.4%[1]), efficacy of the CRM,[3,4] and healthcare efficiencies.[1,13,19-21] We bring together investigators of multiple disciplines – including emergency medicine (academic and community practice), neurology, otolaryngology, general medicine, behavioral science, and implementation science -- with the goal of helping physicians address a problem they have declared to be a top priority for decision support and which is associated with high frequency of unnecessary testing and low frequency use of evidence-based practices.[1,13,19-22] It is expected that this project will have a direct positive impact on the effectiveness and efficiency of care for BPPV presentations, and others.

This work is licensed under a Creative Commons Attribution-NonCommercial-NoDerivatives 4.0 International License.

Frontline physicians want support for vertigo. A survey of ED physicians about priorities for the development of clinical decision support (1,150 respondents) ranked vertigo as the #1 topic in adult ED presentations.[22] The lowest hanging fruit in the opportunity to achieve meaningful improvements in dizziness presentations is BPPV. BPPV is common, and readily identifiable and treatable at the bedside. No laboratory or imaging studies are needed, and in fact these are explicitly discouraged in guideline statements.[4] ED physicians have strongly advocated for the use of BPPV processes (even stopping an ED-based trial for ethical reasons given the effect size at interim analysis),[5] and our survey (preliminary studies) indicates high demand for BPPV intervention.

2.2 **Supporting Data**

Two Evidence-Based Guidelines support the Dix-Hallpike Test (DHT) and the Canalith Repositioning Maneuver (CRM) to diagnose and treat BPPV. Evidence-based guidelines supporting the DHT and CRM were published in 2008 by the American Academy of Otolaryngology-Head and Neck Surgery and the American Academy of Neurology.[3,4] Additional systematic reviews also support the DHT and CRM.[6,7,12,23,24] The primary RCTs demonstrate the resolution of BPPV symptoms (outcomes measured at 1 day to 4 weeks) in patients treated with the CRM.[9-11,25,26] In these studies, 61% to 80% of treated patients had resolution after just one treatment compared with 10% to 48% of untreated patients. These effect sizes translate in to a number-needed-to treat ranging from 1.4 to 3.7, which is among the most substantial effects achievable in clinical medicine. In the study assessing outcome at 24-hours, 80% of treated patients were cured versus only 10% of controls.[25] Substantial benefit has also been demonstrated in RCTs from primary care settings.[5,8]

The potential risks to patients include risk of the benign paroxysmal positional vertigo (BPPV) processes (i.e., Dix-Hallpike test [DHT] and canalith repositioning maneuver [CRM]), and risk that the intervention could reduce use of other tests and result in missing a diagnosis that would have otherwise have been made. The intervention is directed at physicians and includes information and instruction of guideline concordant processes which physicians can use at their discretion. The BPPV processes are guideline supported. Guidelines state that complications of BPPV processes are generally mild and self-limited (e.g., nausea or vomiting) and that “serious complications from the processes have not been identified in multiple RCTs.”[4] However, potential risk of the processes exists if the BPPV processes are applied in patients in whom the movements are not safe or tolerable. For example, patients with cervical spine instability would be at risk of spine injury from lying down with the head extended back. Also patients with severe congestive heart failure may develop shortness of breath when lying flat. These types of patients were unlikely to have been included in prior RCTs (although such exclusions are not explicitly stated). It is also possible that the intervention could change physician ancillary test ordering practice patterns. For example, a physician who typically orders a head CT in patients with dizziness may order these tests less frequently after learning to identify and treat BPPV. If providers reduce their typical test ordering, it is possible that a diagnosis that may have been made if the

This work is licensed under a Creative Commons Attribution-NonCommercial-NoDerivatives 4.0 International License.

test was ordered would be missed. This is unlikely, and discussed further in sections 10.1-10.2 (risks and protections against risks).

3 STUDY DESIGN

This is a partnered best-practice implementation study, meaning that the local providers will be engaged in the intervention components. The testing design method is a stepped-wedge, randomized clinical trial of a multi-faceted educational and care-process based intervention designed to improve the guideline-concordant care of patients with BPPV in the emergency department. The unit of randomization is the hospital. After an initial observation period, the six hospitals will undergo the intervention in five waves (the two closely integrated hospitals will be paired). The order that each hospital receives the intervention will be randomized. The intervention will be provided as a complete package during the month each hospital is randomized to. Further detail about the intervention is given in Section 4.

The current design will allow us to estimate the amount of change in the use of guideline concordant BPPV care processes that is associated with the delivery of the intervention. Each hospital will serve as its own control providing pre and post observations. Since this is stepped wedge, some hospitals will contribute many more pre-intervention observations and this will allow us to observe for underlying secular trends to ensure that changes observed in care processes are attributable to the interventions.

The focus of the intervention is on health systems and frontline medical providers, however the observations and outcomes are derived from the medical records of identified cases of dizziness in the included emergency departments. As patients are not directly intervened upon, and the intervention is to improve guideline concordant care, we are seeking a waiver of informed consent for the collection of patient level outcomes from the medical record, along with a waiver of documentation of informed consent for brief phone interviews and SMS and online surveys with patients.

Health care providers involved in the study will be informed of the voluntary nature of this research and will not be required to use any tools or attend any educational sessions provided by this team. See companion application “Medical Provider Component” UM IRB HUM 00113988.

4 SELECTION AND ENROLLMENT OF SUBJECTS

Dizziness visits (Section 4.1), The dizziness visits are the unit of analysis for the primary measure of use of BPPV processes by the health care providers.

4.1 <u>Inclusion Criteria for Dizziness Visit Medical Record Review and Data Abstraction</u>

4.1.1 Age of 18 or greater

This work is licensed under a Creative Commons Attribution-NonCommercial-NoDerivatives 4.0 International License.

4.1.2 ED patient seen at one of six full service non-freestanding Emergency Departments in Nueces County, Texas

4.1.3 Principal dizziness case: the triage reason for visit is a dizziness symptom OR a dizziness symptom is one of first three listed complaints in physician medical record OR a dizziness diagnosis (e.g., dizziness or vertigo NOS, BPPV, vestibular neuritis) is recorded as one of the first three final ED diagnoses.

4.2 *Exclusion Criteria*

4.2.1 Prisoners

4.2.2 Cognitively impaired adult (defined for study purposes as notation of mentally retardation or similar diagnosis within the chart)

4.3 *Study Enrollment Procedures*

4.3.1 Hospital inclusion **-** Data collection will commence in the pre-intervention period and at that point, the hospitals will be engaged in research

4.3.2 Identification of Cases

Cases of dizziness presentations will be captured by active and passive surveillance. Study abstractors will be blinded to randomization periods and groups. For active surveillance, ED logs will be screened for dizziness terms (e.g., dizziness, vertigo, imbalance, spinning, lightheadedness, nausea common misspellings or similar terms) as the patients' reasons for visiting the ED. Passive surveillance will be performed by screening a ICD-9/10 hospital database for dizziness and vestibular ICD-9/10 codes. We do not limit the population to patients receiving a BPPV diagnosis for the following reasons: the likelihood of diagnostic misclassification,[17,21] the high frequency of cases receiving symptom only diagnoses (see preliminary results), and diagnostic bias introduced by the intervention during the post-intervention phase (e.g., providers may be more likely to use BPPV diagnoses after the intervention).

4.3.3 Screening and enrollment

All cases meeting the inclusion criteria and the definition of principal dizziness visit will undergo data abstraction and will be included in the overall trial database. We will collect monthly counts of the number of charts with any screening dizziness terms at each site.

4.3.4 Consent/assent procedures – patient level data collection

Patients will be receiving routine clinical care at the local emergency departments and we have sought a waiver for review and collection of data from the medical charts. For follow up telephone surveys of patients in the emergency department,

This work is licensed under a Creative Commons Attribution-NonCommercial-NoDerivatives 4.0 International License.

we will use a structured script to provide information about the study and get informed consent to ask brief questions about the performance of BPPV care processes in the emergency department (Appendix A). For the SMS and online surveys, patients will be asked to opt-in if they are willing to complete the study questions (Appendix A).

4.3.5 Intervention group assignment
Patients will be assigned to either the intervention or control group based on the time and hospital they present to for their dizziness care. If the hospital has received the intervention and is in the post-intervention period – they will be in the intervention group, otherwise they will be considered control patients (pre-intervention). The hospitals will receive the intervention at a randomly assigned time point as described below in Section 5.

5 STUDY INTERVENTIONS

5.1 Interventions, Administration, and Duration

The overall educational intervention is delivered at the hospital level. Table 5.1 provides an overview of the hospital level interventions that occur as part of the trial. We describe each in greater detail in sections 5.1.1- 5.1.5. Attendance by medical providers will be tracked to determine utilization.

Table 5.1. Multi-faceted Preliminary Intervention Overview.

**Implementation strategy components**	**Component Description**
1. Local champions	Local champions (ED providers) will be recruited and trained in BPPV testing and diagnosis. Each will participate in CME session, follow-up sustainability session, and commit to help during routine care.
2. Educational sessions. Interactive and hands-on sessions.	Sessions will review BPPV mechanisms and evidence, utilize videos, include hands-on demonstration, and address barriers from aim 1
3. Decision aid. Multi-media web-based decision aid application	Includes high yield text and videos on the BPPV processes and characteristic exam findings. Audit and Feedback – split out as own row. Will also include individual and group level feedback on dizziness care process delivery over time.
4. Referral resource	Readily available list of outpatient experienced BPPV providers accepting referrals
5. Follow-up educational sessions	Sessions, led by local champions, to facilitate adoption, implementation, and sustainability.

This work is licensed under a Creative Commons Attribution-NonCommercial-NoDerivatives 4.0 International License.

6. Partnered development and other resources	Other resources, identified by and developed by the local medical providers may be provided.

5.1.1 Champion Development

Local ED physician champions will be recruited for each hospital system. These champions will serve as the point person of contact for the local ED physician groups, will receive more focused training on BPPV prior to the CME educational sessions, will be a key participant (instructor) at the CME session, will lead follow-up educational sessions, and be available for questions as needed.

5.1.2 Interactive High Yield, Hands-on Education Session (CME session).

An evidence-based educational session will be developed to be presented by Drs. Kerber, Meurer, and local champions to the Corpus Christi ED physicians, physicians assistants, and residents and will be used to introduce the decision aid. Common barriers will be addressed along with suggestions for overcoming barriers. For example, we anticipate that one barrier in the ED will be small exam gurneys. Throughout the session, videos will be used to enhance learning. Videos will demonstrate the DHT and CRM, the characteristic BPPV nystagmus, and other eye movements that can be misinterpreted as BPPV nystagmus (e.g., eyelid blinks, voluntary movements, and nystagmus patterns of vestibular neuritis, or a central lesion). Descriptions of factors that could lead to misclassification of BPPV cases based on the DHT will be provided, such as extremely slow movements or basing the test interpretation on symptoms rather than the characteristic pattern of nystagmus. A specific part of the session will focus on risks of the BPPV processes and dangerous mimickers of BPPV (See Human Subjects Section). It will be made clear that the processes should be considered contraindicated in patients with known or suspected cervical spine instability until the spine is cleared. Interactive techniques will be incorporated to maximize the probability of outcome success by adhering to principles of adult education: delivering content in a learner-centered, active format, relative to the learner's needs, which is simultaneously engaging and reinforcing.[26] The sessions will target increasing self-efficacy (perception of one's abilities) and outcome expectancy (belief that a behavior will lead to the desired outcome). High outcome expectancy is associated with an increased likelihood of performing a behavior.[27] Hands-on training for the DHT and CRM will also be developed as part of the presentation. Physicians will pair up at tables and perform the DHT and CRM under instructor guidance (i.e., investigators and champions). Models of the semicircular canals will be used to facilitate understanding the basis for the processes. CME credit and a modest incentive $50 will be offered to encourage medical provider attendance. Individual attendance will be recorded. To promote the widest exposure of the educational sessions, the sessions will also be video recorded and made available for post-intervention physicians and medical providers to review on their own time. In addition, individual or small group sessions will be offered for providers not

This work is licensed under a Creative Commons Attribution-NonCommercial-NoDerivatives 4.0 International License.

able to attend the primary sessions. To improve later presentations, the project team will review recordings of CME sessions. .

5.1.3 Web-based, multi-media real-time decision aid.

We have developed a web-based application in accordance with the findings from physician interviews and environment barrier assessments (www.dizztinct.com). This aid is a tool providers will be able to efficiently use at the point of care. Extensive collaborations with our behavioral scientists and technology developers informed the content and structure of this aid. We plan for providers to be able to use the aid in less than 10 minutes, though it will also include additional resources and information so that more details are available for interested providers. The aid uses videos and includes brief high yield narration, video instructions on the DHT and CRM, and video demonstrations of positive and negative test results. The videos demonstrate dangerous signs of central nervous system disorders. The aid also highlights potential risks of the processes and BPPV mimickers. Additionally, it states that known or suspected cervical spine instability is a contraindication until the spine is cleared.

The aid will be password protected to each individual’s identity.

The aid will also contain tailored data on the individual’s performance on dizziness care processes. The proportion of eligible dizziness cases receiving the DHT (primary BPPV diagnostic endpoint) and the CRM (secondary BPPV therapeutic endpoint), will be plotted over time. This will be graphically summarized to demonstrate the performance of all health care providers (anonymized to user) at the site over time, and the individual data point corresponding to the user will be noted in a monthly email and within the website.

The aid will also be available in a mobile version for smartphones and tablets

5.1.4 Referral resource

Because our main goal is to get the right treatment to the right patient within a reasonable time frame, we will also establish a list of providers in the community who evaluate and treat BPPV. The goal of this system is to provide ED physicians with a more informed route of referral to appropriate community providers for BPPV treatment. Many providers who see patients with BPPV or probable BPPV would like to make a specific referral to another provider for evaluation, treatment, or subsequent assessment. Our previous survey work of community ED providers found this to be a popular option. To establish this resource, providers in the community who evaluate and treat BPPV will be identified, covering a variety of insurance/payment options. Physical therapists, particularly those with training in vestibular therapy, are likely to be an important resource. We have already been in contact with PTs in the community in this regard. A system will be established to track use of referrals. In general, we will use the existing hospital methodology

This work is licensed under a Creative Commons Attribution-NonCommercial-NoDerivatives 4.0 International License.

for referrals, however we may be able to augment this with a list of local providers who are willing and able to quickly see and treat these patients.

5.1.5 Follow up Educational Sessions

Follow-up maintenance sessions will also be developed for the time period after the first CME session. These sessions will be used to facilitate adoption, implementation, and sustainability. The format of follow-up sessions will be case-based. Providers will be encouraged to express successes and failures. Feedback on BPPV processes utilization will be presented. These sessions may be led by the designated local champion, an interested local medical provider, or the study team. These sessions may be in person or by telephone or web conference.

5.1.6 Partnered adaptations of other Resources Identified by Local Medical Providers

This is a partnered implementation project, and based on our engagement with the providers during the project we expect to learn even more about provider preferences and needs based on provider feedback. Thus, in addition to the steps in Section 5.1.1 – 5.1.6., the intervention components can be adjusted/adapted based on provider feedback and requests, consistent with the approved protocol for this research. For example, it is possible that providers may request addition resources or patient specific materials. If they make such a request, we will help them in finding/developing/implementing such other resources. We, as the researchers, will not generate such materials ourselves without proper regulatory approval.

(Update 12/7/2017) In the course of engaging with providers, they requested an additional website/app component to prospectively collect information related to safety in discharging ED dizziness patients home (Appendix C). We worked with providers to develop a list of items and data collection form. The list of items includes the BPPV specific items and additional items that largely relate to the possibility that the patient might have a stroke as the cause or is high risk for stroke in the short term. Both of these components fit well with our overall intervention which emphasizes both the features of BPPV and also findings that suggest an alternative cause such as stroke. The addition of this component could serve to: 1) increase exposure to the BPPV related items (an additional resource providers may seek out), 2) enhance data capture in a subsample of visits, and 3) build engagement/collaboration with local providers. The new prospective data collection could enhance implementation fidelity measures because this prospective provider-entered data enables an assessment of the consistency of medical record documentation of BPPV assessments. In addition, the new prospective data regarding safety items should enhance secondary analysis regarding the 90-day stroke rate by informing factors that predict stroke (in the subsample with data collected).

In the form, we separate the BPPV items from the safety items to enforce that the BPPV items are established clinical guideline items but that the safety items are not. The form also explicitly states that the safety items are for data collection purposes only. There is no

This work is licensed under a Creative Commons Attribution-NonCommercial-NoDerivatives 4.0 International License.

current society clinical guideline regarding safety in discharge of ED dizziness patients. To develop these items, we reviewed the medical literature regarding factors previously shown to predict stroke. The items were then created/edited by our team of investigators (neurologists, emergency medicine, otolaryngology, general medicine) and local ED providers. If the analysis of these safety items indicates they are accurate in discriminating stroke, then future studies (requiring separate IRB application) may be done to test the effect of the list on outcomes in dizziness ED cases (e.g., subsequent stroke event, length of stay in the ED, test utilization).

The form will be available on the website/app. Providers can voluntarily complete the form at the point of care. Data can be entered electronically or paper forms can be printed, completed, and inserted into a lock-box in the ED. CHRISTUS Spohn Shoreline already has an available lock-box for several other projects that use a similar method for data collection.

5.2 Randomization

The overall study period will be approximately 18 months. After the 4-month pre-intervention period, the sites will receive the intervention in 5 waves. (Two closely integrated hospitals will receive the intervention at the same time.) The order will be determined by using a random number generator.

5.3 Concomitant Interventions

The community will be closely monitored for any large secular trend (i.e. a health insurer or malpractice insurer based intervention on the care of dizziness in the emergency department.) The primary analysis will remain unchanged, but secondary analyses will be conducted to estimate the impact of this change within hospitals that have and have not received the intervention by the time of the event.

6 CLINICAL AND LABORATORY EVALUATIONS

6.1 Timing of Evaluations – Patient level

6.1.1 Event 1: Emergency Department Visit

The initial emergency department visit for a principal dizziness complaint will be identified using the screening procedure noted above. Data will be abstracted from the chart regarding the primary outcome, demographics, ED length of stay, imaging utilization, medications utilized and ED disposition. If the patient is hospitalized, additional data regarding hospital length of stay and procedures will be collected from administrative data.

6.1.2 Event 2: Telephone interview, or electronic survey via SMS or website

A brief, structured interview will be conducted to determine whether a BPPV care process (DHT, CRM, or referral) occurred while the patient was in the emergency depart-

This work is licensed under a Creative Commons Attribution-NonCommercial-NoDerivatives 4.0 International License.

ment. A random subset of cases will receive a phone call within 3-5 days of the ED visit. Three attempts will be made. The caller will briefly describe the research study and seek verbal informed consent using a script to ask the brief survey (appendix A). If voicemail is available for the caller, we will leave a message with contact information for study personnel. The script includes language emphasizing that BPPV care processes are not recommended for every patient with dizziness and that this phone call has nothing to do with whether they should or should not have received a BPPV care process. The voicemail script will also give the patients the option to complete an electronic version of the survey either via text message or an online survey link (Qualtrics). Patients will be instructed that they can reply to the phone call with a text message to initiate the SMS survey. The online survey address will also be included in the voicemail (appendix A).

6.1.3 Event 3: Stroke outcome assessment

The Brain Attack Surveillance in Corpus Christi (BASIC) Project is a concurrent, epidemiological study that is identifying all cases of stroke in Nueces County, Texas and is approved by the University of Michigan and local IRBs (HUM00041536). Patients identified as part of the trial aged 45 and above will be cross referenced and linked (using medical record number, name, gender, age and other demographic data) across the BASIC database by study staff at 90 days (+/- 14) after the index ED visit. Cases with neurologist validated stroke will be identified and the BASIC patient ID number will be entered as a database field in the trial database to allow for later characterization of these cases using the full BASIC dataset (including 90-day outcome data). Other data captured in the trial database from the stroke event will include the date of the event. Identity linkage to search for individuals admitted to other Nueces County hospitals for stroke will occur routinely, approximately quarterly. We will separately apply for data from the Texas Department of Health so we can identify participants who died for non-stroke reasons (stroke deaths are already captured as part of BASIC); the purpose of this is to ensure accurate identification of the population at risk for stroke during the follow up period by censoring subjects who have died on the day of death.

6.1.4 Event 4: End of surveillance of individual case

At the end of 90 days, no additional evaluation of the patient level data will occur. Identifiers will remain in the study database until the end of the overall trial in order to identify patients with repeat visits. At the end of the study, a linkage will remain to the coded identifier used as part of the BASIC study for cases with neurologist validated stroke. This linkage will allow elements of stroke (severity, 90-day outcome following stroke) collected by BASIC to be summarized at the individual patient level. Relevant unique data fields will become part of the permanent trial dataset. All linkages to identity will be destroyed one year after the publication of the primary outcome paper. The permanent trial dataset will meet the criteria for a de-identified dataset.

6.2 **Timing of Evaluations – Hospital Level**

This work is licensed under a Creative Commons Attribution-NonCommercial-NoDerivatives 4.0 International License.

6.2.1 Screening and Enrollment

On a weekly basis, each emergency department log will be reviewed for primary dizziness visits. In addition, a list of ED reason for visit and diagnosis codes meeting the inclusion criteria above will have medical records identified and evaluated. Cases meeting the inclusion criteria will comprise the patient population of the study. Patient level data will be accessed in the participating hospitals.

7 MANAGEMENT OF ADVERSE EXPERIENCES

The definitions of adverse events (AEs), and serious adverse events (SAEs) are given below. It is very important that all staff involved in the study are familiar with the content of this section.

7.1 Definition of Adverse Event and Serious Adverse Event and Scope of AE reporting

An adverse event (AE) is any unfavorable and unintended sign (including a clinically significant abnormal laboratory finding), symptom, or disease temporally associated with the use of a medical treatment or procedure regardless of whether it is considered related to the medical treatment or procedure (attribution of unrelated, unlikely, possible, probable, or definite). Each AE is a unique representation of a specific event used for medical documentation and scientific analysis.

A serious adverse event (SAE) is an AE that is fatal or life threatening, is permanently or substantially disabling, requires or prolongs hospitalization, results in a congenital anomaly, requires intervention to prevent permanent impairment or damage, or any event that the treating clinician or internal medical monitor judges to be a significant hazard, contraindication, side effect, or precaution. Reporting serious adverse events (SAEs) are based on the guidelines of the International Conference on Harmonization (ICH).

For the purposes of the trial, where the intervention is targeted on providers, we will only capture patient level AEs and SAEs that are possibly, probably or definitely related (see section 7.5) to the performance of BPPV care processes (Dix-Hallpike test or Canalith Repositioning Maneuver).

7.2 Unexpected (Unanticipated) Adverse Event

An unexpected AE is defined as an event that is not anticipated or known to occur as an established risk of either the study intervention or the subject's underlying medical condition. Expected events include, but are not limited to, those specifically identified and described or listed in the study protocol and/or informed consent document.

7.3 Severity of an Adverse Event

'Severity' is not the same as 'serious.' Serious is based on patient/event outcome or action criteria usually associated with events that pose a threat to a patient's life or health. The term 'severe' is often used to describe the intensity (severity) of a specific event (as in mild, moderate, severe

This work is licensed under a Creative Commons Attribution-NonCommercial-NoDerivatives 4.0 International License.

myocardial infarction); the event itself, however, may be of relatively minor medical significance (such as severe headache). Seriousness (not severity) serves as a guide for defining regulatory reporting obligations. Most AEs include clinical criteria that describe patient/event outcomes or indicated interventions to more clearly substantiate seriousness.

7.4 Classification of an Adverse Event

All adverse events occurring within 24 hours of treatment and all serious adverse events occurring during study participation will be documented on the AE case report form. Adverse events will be documented using the NCI Common Terminology Criteria for Adverse Events Version 3.0 (CTCAE). The CTCAE provides descriptive terminology that will be used for recording and reporting adverse events that occur in the trial. The CTCAE provides a grading (severity) scale for each AE term and AEs are listed alphabetically within categories based on anatomy or pathophysiology. The CTCAE (v 3.0) displays Grades 1-5 with unique clinical descriptions of severity for each AE based on this general guidance:

- Grade 1: Mild AE
- Grade 2: Moderate AE
- Grade 3: Severe AE
- Grade 4: Life-Threatening or Disabling AE
- Grade 5: Death related to AE

Note: Severity is not equivalent to seriousness. A serious adverse event (SAE) would be any event in category 4 or 5, and any event in category 3 that required or prolonged hospitalization.

Not all grades are appropriate for all AEs. Therefore, some AEs are listed with fewer than five options for Grade Selection i.e., Grade 5 (Death) is not appropriate for some AEs and therefore is not an option.

7.5 Relationship to Study Treatment

The principal investigator is responsible for designating, at the time an AE is reported, how likely it is that the AE was caused by the study intervention. These decisions (classification of relatedness) will be reviewed by the Independent Medical Monitor. This determination requires clinical judgment, but for purposes of this study an algorithm is used to help the investigator provide reporting that is as objective as possible and consistent with reporting across the trial.

- *Not related:* The temporal relationship between treatment exposure and the adverse event is unreasonable or incompatible and/or adverse event is clearly due to extraneous causes (e.g., underlying disease, environment). NOTE: The trial will not collect or tabulate AEs that are in this relationship category.

- *Unlikely*: May have reasonable or only tenuous temporal relationship to intervention. Must meet both of the following conditions:
  - Could readily have been produced by the subject’s clinical state, or environmental or other interventions.
  - Does not follow known pattern of response to intervention.

This work is licensed under a Creative Commons Attribution-NonCommercial-NoDerivatives 4.0 International License.

- NOTE: The trial will not collect or tabulate AEs that are in this relationship category.

- *Possibly*: Must meet any 2 of the 3 following conditions
  - Has a reasonable temporal relationship to intervention.
  - Could not readily have been produced by the subject's clinical state or environmental or other interventions.
  - Follows a known pattern of response to intervention.

- *Probably*: Must meet all 3 of the following conditions
  - Has a reasonable temporal relationship to intervention.
  - Could not *readily* have been produced by the subject's clinical state or have been due to environmental or other interventions.
  - Follows a known pattern of response to intervention.

- *Definitely*: Must meet all 3 of the following conditions
  - Has a reasonable temporal relationship to intervention.
  - Could not *possibly* have been produced by the subject's clinical state or have been due to environmental or other interventions.
  - Follows a known pattern of response to intervention.

7.6 Adverse Event Recording and Reporting

All serious adverse events (SAEs) meeting the study definition will be reported to the IRB within seven days of discovery. All other AEs meeting the study definition will be entered into the database and reported to IRBs at the next scheduled continuing review. All SAEs and AEs will be reported at the scheduled meetings of the DSMB.

7.7 Expected / Anticipated Adverse Events Associated With Dizziness Care Processes

The performance of dizziness care processes may cause adverse events. These will occur during the process of routine clinical care in the emergency department.

Common adverse events – patient level

- Nausea
- Vomiting
- Neck pain / discomfort
- Note: worsening of dizziness is not considered an adverse event as the dizziness is the qualifying condition for the trial, and BPPV care processes can be expected to elicit or exacerbate dizziness and nausea, at least briefly.

Uncommon adverse events (theoretically could be associated with performance of BPPV care processes) – patient level

This work is licensed under a Creative Commons Attribution-NonCommercial-NoDerivatives 4.0 International License.

- Neck injury
- Spinal cord injury
- Vascular injury (carotid or vertebral dissection; Note: carotid or vertebral dissection causing stroke are likely to be pre-existing conditions.)
- Fall with trauma (head, neck, back)

8 STATISTICAL CONSIDERATIONS

8.1 General Design Issues

The primary objective is to evaluate, in a community ED setting, the effect of the multi-faceted, theory-based educational intervention and implementation strategy on the use of the Dix-Hallpike test and Canalith Repositioning Maneuver in ED dizziness visits. The secondary objectives are to evaluate variation in BPPV process utilization at the provider level, care processes (ED length of stay, neuroimaging, inpatient hospitalizations), and health care costs.

The primary hypothesis is that patients seen at hospitals that have received the intervention will have a higher likelihood of receiving a BPPV care process (i.e., DHT, CRM) compared to patients seen at hospitals that have not received the intervention.

Validity of primary endpoint: The primary endpoint has been rigorously evaluated in our preliminary work. The documentation of performance of BPPV care processes is rare, and in discussion with physicians in emergency medicine practice, the rarity of documentation is well reflected in clinical practice: BPPV care processes are rarely used and rarely documented.

The secondary objective evaluates the performance of any BBPV care process, including performance of the DHT, or CRM or a referral to a BPPV care provider. We hypothesize that patient visits occurring at hospitals that have received the intervention will have a significantly different rate of having any of the BPPV care process endpoints (documentation of DHT or CRM or outpatient referral for BPPV evaluation to a BPPV provider) associated with the ED care compared to patient visits occurring at hospitals that have not received the intervention.

The main safety hypothesis is that there will be no difference in the rate of index visit and short-term (90 day) stroke within the dizziness population aged 45 and older, in patients seen in EDs that have received the intervention versus patients seen in EDs that have not received the intervention.

8.2 Endpoint

8.2.1 The primary endpoint is measured at the individual patient level, and is the presence of documentation of either the Dix-Hallpike test, the Epley Maneuver (or CRM) (since the first step of the CRM is the diagnostic maneuver).

This work is licensed under a Creative Commons Attribution-NonCommercial-NoDerivatives 4.0 International License.

8.2.2 Secondary endpoints: 1. Documentation of Dix-Hallpike alone in the ED. 2. Documentation of CRM during ED visit. 3. Outpatient referral to a BPPV provider (ENT, neurologist, physical therapist).

8.2.3 Safety secondary endpoint: 90-day cumulative incidence of stroke in patients aged 45 and above following initial ED discharge home visit for dizziness.

8.2.4 Additional exploratory endpoints: Stroke at index ED dizziness visit, ED length of stay in hours, neuroimaging utilization, and inpatient hospitalization utilization.

8.3 Sample Size and Accrual

The trial will start with an initial no intervention period of approximately 4 months followed by randomized staggered intervention with a new hospital entering approximately every 2 months, finalized by approximately 4 post-intervention months will result in the approximately balanced number of 867 visits occurring without intervention and 933 visits occurring under (post) intervention. This calculation assumes the average anticipated total patient visit rate of 100 patients per month. Based on our pilot studies and the literature we expect the DHT or CRM procedure to be done in 5% patients before the intervention. With the expected number of visits calculated above, we will be able to detect the increased DHT or CRM rate of 9% and above with 90% power by a two-sided test at the significance level of 5%. We expect a much bigger difference of 5% vs. 20% pre- vs. post-intervention DHT/CRM rates, respectively. Under this expected difference, we will have the power exceeding 99%. In fact, an order of magnitude smaller visit rate of 10.4 patients per month would be sufficient for 90% power under the anticipated difference. The reserves of power will be used to provide more power to fine-tune the multivariate mixed regression models and associated secondary analyses.

Patients: Each month, approximately 100 patients with dizziness seek care at the six hospital associated-emergency departments in Nueces County, Texas. Over an 18-month period we anticipate a total sample size of approximately 1,800. Approximately half of the 1,800 patient visits will occur prior to the intervention in the overall study. As dizziness volume can vary widely both within and across emergency departments we have provided maximum sample sizes for each site that are 200% of our initial estimates. After evaluating initial accrual, a higher number of ED visits for dizziness met our inclusion criteria, and as such we are now providing increased maximum sample sizes. Our revised total expected enrollments will be 6800 with a revised maximum of 10,800. We believe the higher than normal number of visits likely relates to a lower threshold for inclusion in the current study than in the study used for preliminary estimates, an increase in dizziness presentations to the ED now compared with time of preliminary estimates, and the closing of one of the EDs (Spohn Memorial) resulting in higher volumes per ED.

Table 8.3 – Anticipated and Maximum Enrollment by Site and Type

This work is licensed under a Creative Commons Attribution-NonCommercial-NoDerivatives 4.0 International License.

Site	Expected Patients	Maximum Patients	Revised Expected	Revised Maximum
Spohn Shoreline	400	800	1800	2800
Spohn Memorial	400	800	800	2000
Spohn South	200	400	1400	2000
CCMC – Doctors	350	700	1000	1750
CCMC – Bay Area / Heart	300	600	1200	1500
CCMC - Northwest	150	300	600	750

.

8.4 Data Monitoring

The trial does not employ formal efficacy or futility stopping rules. As a cluster delivered educational intervention to several hospitals, formal stopping boundaries for efficacy or futility are not appropriate.

The Independent Medical Monitor will review primary endpoint data by group (See also Section 11 and Appendix B for more details about the IMM). The principal investigators will review overall primary outcome data and will remain blinded to performance by group. In addition, the main safety endpoint (90-day stroke rate) will be included for reporting to the investigators and the IMM; it will be further classified as immediate diagnosis (at time of index ED primary dizziness visit) or delayed diagnosis (at some point during following 90 days). A brief narrative will be provided with each stroke diagnosis, based on the linked record from the BASIC study (i.e. 50 y/o female with middle cerebral artery stroke 85 days after ED primary dizziness visit).

A summary of SAEs and AEs meeting the DIZZTINCT definition for inclusion will also be routinely monitored by the PIs and the IMM.

Data quality will be assessed by routine monitoring by the study project monitor, along with review of the interview data that assesses patients directly for the performance of BPPV care processes.

This work is licensed under a Creative Commons Attribution-NonCommercial-NoDerivatives 4.0 International License.

8.5 Data Analyses

The intervention is delivered to hospitals. Intervention is a binary variable with two levels, pre-intervention (no intervention), post-intervention (under intervention).

The primary analysis will use binary logistic regression and will include covariates for hospital, month (to handle secular trends), and intervention (see below). For a set of new patient visits, the binary random variable DHT/CRM/referral yes/no will serve as the primary response. Patient visits will be supplied with patient-, hospital- and provider-level covariates as well as the calendar time variable modeling the secular trend, and the intervention yes/no variable measuring whether the visit occurs under intervention or not. To take hospital- and provider-specific unmeasured features into account, hospital and provider categorical variables will be included in the analysis. Due to the fact that the number of hospitals and providers is much smaller than the number of patient visits, adjusting for hospital and provider effects by way of categorical variables will not lead to bias. Secondary analyses will explore alternative approaches using random effects (Gaussian) models. A two-sided model-based test for the intervention variable will be used to test the primary hypothesis at the significance level of 5%.

The secondary analysis (safety) will numerically summarize the 90-day stroke rate - cumulatively and stratified for stroke diagnosed on the index dizziness visits and for post index visit strokes (delayed diagnosis) in patients seen at EDs with and without the intervention. This is anticipated to be very rare. The intervention does not target improving stroke diagnosis. However, evaluating both the index visit stroke diagnosis rate and the delayed diagnosis rate should allow for determination of major changes. We anticipate the index visit stroke diagnosis rate to be approximately 2% and the delayed diagnosis rate approximately to be 1%.

9 DATA COLLECTION, SITE MONITORING, AND ADVERSE EXPERIENCE REPORTING

9.1 Records to Be Kept

Data will be abstracted from the medical record into the subject's research chart. In addition, the full ED record (nursing notes, physician notes, medication administration, laboratory testing, imaging results) will have patient level identifiers redacted and will be scanned and uploaded into the study database. The date of visit will also be redacted from the scanned charts. Identifying information will be collected for each individual in the study (medical record numbers at each of the participating hospital, name, date of birth, address and contact information). This will be used to coordinate the 3-5 day telephone calls and electronic surveys and also to track subjects to determine if they have had a stroke and also to determine if they have additional ED visits for dizziness. We will maintain a link between the study dataset and the patient identifiers to monitor for recurrent events and link to stroke. This linkage file will be retained until the end of data analysis.

This work is licensed under a Creative Commons Attribution-NonCommercial-NoDerivatives 4.0 International License.

Limited access, HIPPAA compliant databases on secure servers will be utilized for all data storage and collection.

9.2 Role of Data Management

A subset of ED charts will undergo additional review to assess data quality. A second assessor blinded to the intervention group assignment (based on hospital), will re-code every single trial chart for the primary outcome. A study investigator who is also blinded to whether that visit occurred before or after the intervention occurred at that hospital will adjudicate cases where the final outcome is discordant between the blinded outcome assessor and the initial abstraction. Outcomes classified as "Not Sure" will also be reviewed by the PIs blinded to the date of the event. Range checks and content validation will be utilized in the database to ensure high quality data entry.

9.3 Quality Assurance

Source documents will be uploaded for remote verification as noted above in section 9.1. Based on the results of the additional review, retraining of the abstractors will occur if issues with accuracy arise.

9.4 Adverse Experience Reporting

For SAEs, the data entry must take place within 24 hours of discovery of the event. SAEs attributable to BPPV (trial AE definition) care processes will be reported to all relevant IRBs within seven days of discovery. The study database will have forms for entry of all adverse events.

10 HUMAN SUBJECTS

In general, the research team will adhere to the following principles:

- We will protect and safeguard HIPAA defined Protected Health Information.
- We will respect the confidentiality of the medical providers and health care facilities.
- There will not be public disclosure of identifiable medical provider performance.
- There will not be disclosure or publication of identifiable hospital level performance.
- Identities (patient, provider, and hospital) will be coded in research databases with the linkage file being removed after data analysis.

10.1 Risks

10.1.1 Data risks

The main potential risk to patients in this study is breach of confidentiality of medical records and survey responses that could result in psychological distress. The likelihood of this risk is estimated to be rare. The seriousness to the subject is estimated to be low. We have methods in place to prevent this occurrence. Data obtained for participants will be

This work is licensed under a Creative Commons Attribution-NonCommercial-NoDerivatives 4.0 International License.

held strictly confidential in locked facilities and password protected computers. Trained abstractors will enter pertinent information into the database using REDCAP or similar secure online database. Each abstractor is restricted to only the data they collect or have a need to access.

Paper forms of the provider checklist will be entered into the secure REDCAP database. PHI will be redacted from the scanned versions that will be retained in the database. The online version of the provider checklist will not collect PHI. Checklists filled out online will be linked back to visits when applicable in our database by determining which provider filled it out and the time that a relevant, included dizziness visit occurred on that day.

10.1.2 BPPV Care process risks

The BPPV processes are guideline supported. Guidelines state that complications of BPPV processes are generally mild and self-limited (e.g., nausea or vomiting) and that "serious complications from the processes have not been identified in multiple RCTs." However, potential risk of the processes exists if the BPPV processes are applied in patients in whom the movements are not safe or tolerable. For example, patients with cervical spine instability would be at risk of spine injury from lying down with the head extended back. Also patients with severe congestive heart failure may develop shortness of breath when lying flat. These types of patients were unlikely to have been included in prior RCTs (although such exclusions are not explicitly stated).
It is also possible that the intervention could change physician ancillary test ordering practice patterns. For example, a physician who typically orders a head CT in patients with dizziness may order these tests less frequently after learning to identify and treat BPPV. If providers reduce their typical test ordering, it is possible that a diagnosis that may have been made if the test was ordered would be missed. (See 10.1.3 risk of missed or delayed diagnosis for specific risks to patients and providers).

10.1.3 Risk of missed or delayed diagnosis

It is also possible that the intervention could change physician ancillary test ordering practice patterns. For example, a physician who typically orders a head CT in patients with dizziness may order these tests less frequently after learning to identify and treat BPPV. If providers reduce their typical test ordering, it is possible that a diagnosis that may have been made if the test was ordered would be missed or delayed.

Delayed diagnosis may result in harm depending on what the diagnosis is. However, in clinical practice, patients with isolated dizziness rarely have a serious or life-threatening cause.

This work is licensed under a Creative Commons Attribution-NonCommercial-NoDerivatives 4.0 International License.

10.2 Adequacy of Protection Against Risk

10.2.1 Waiver of documentation of written informed consent for survey procedures –

Patients (Telephone and electronic surveys): The study team will conduct follow up telephone or electronic surveys with patients as described in Appendix A. As the study team will be reviewing ED records retrospectively, it would not be feasible to obtain informed consent at the time of the initial visit. In addition, the phone and electronic surveys are minimal risk to the participants. A consent process will occur via the telephone script or electronic opt-in.

10.2.2 Waiver of informed consent – initial data abstraction

We seek a waiver of consent to screen medical records and to abstract information from subjects meeting the inclusion/exclusion criteria. The risk from medical record review is no more than minimal risk. For study validity, we will need complete case capture. Due to the unpredictable timing of ED dizziness visits, it would not be feasible to obtain prospective informed consent from patients in real time.

10.2.3 Protection from loss of confidentiality / psychological distress

We are mindful of the sensitive nature of patient's medical records and have the utmost concern for the human subjects who will be part of this study and will take steps to ensure protection of confidentiality. Data obtained for participants will be held strictly confidential in locked facilities and password protected computers. All information will be kept in a password protected database such as REDCAP with secure servers used to transfer data between study sites. Computers will be locked or kept with the study team member at all times. Each study team member will be restricted to only the data they collect or have a need to access. We do not anticipate adverse effects to subjects during this study.

10.2.4 Protection against risk from BPPV care processes

The goal of this study is to optimize the appropriate and safe use of the BPPV processes. The risk of the BPPV processes will be described to the local healthcare workforce in the local champion development, the educational hands-on session, the web-based point-of-care decision aid, and at the follow-up educational sessions. It will be expressed that the BPPV processes are contraindicated in patients with known or suspected cervical spine instability until the cervical spine is cleared by the providers. Conditions associated with cervical spine instability (e.g., Down syndrome, severe rheumatoid arthritis, and Paget's disease) will be specifically described. It will additionally be expressed that caution should be taken in any patients who may have increased risk of being intolerant to the BPPV processes, such as those with severe congestive heart failure who may become short of breath while laying down.

This work is licensed under a Creative Commons Attribution-NonCommercial-NoDerivatives 4.0 International License.

10.3 Institutional Review Board (IRB) Review and Informed Consent

This protocol and the telephone consent script(Appendix A) and any subsequent modifications will be reviewed and approved by the IRB or ethics committee responsible for oversight of the study.

10.4 Subject Confidentiality / Data Retention

Patients: In order to link identities to recurrent dizziness visits and strokes within the community, a separate database with subject identifiers and medical record numbers will be maintained for one year after the last subject is enrolled to ensure capture and linkage of outcome events and linkage to stroke events in the BASIC database.
An internal code will be used to link visits to specific hospitals.
All records will be kept in a locked file cabinet or on secure computer systems. Clinical information will not be released without written permission of the subject, except as necessary for monitoring by IRB, the FDA, the NIDCD, the OHRP, the sponsor, or the sponsor's designee. In accordance with NIH regulations, we will create a permanent de-identified, public use database. We will remove linkages to protected health information, hospital identifiers and patient identifiers. The purpose of the retention of this data is for future research and to comply the NIH regulations for datasets created during the conduct of NIH funded research grants.

10.5 Study Modification/Discontinuation

The study may be modified or discontinued at any time by the IRB, the NIDCD, the OHRP, the FDA, or other government agencies as part of their duties to ensure that research subjects are protected.

11 INDEPENDENT MEDICAL MONITOR PLAN

The trial will utilize an independent medical monitor (IMM). The detailed IMM plan is available as an appendix. The target population of the intervention is physicians. The implementation strategy aims to increase the appropriate use by physicians of the guideline supported test and treatment of benign paroxysmal positional vertigo. In the education part of the intervention, we will explicitly describe potential risks and contraindications. Providers will then be able to use their own judgment in identifying the appropriate patients. The reason for using an IMM is the potential risk of performing of BPPV processes inappropriately or inappropriate selection of patients. An additional risk is that providers exposed to the intervention may change their practice regarding test ordering (e.g., less ordering of head CT scans) which could result in missing a diagnosis that may have been made otherwise.

The frequency of IMM meetings will be determined by the IMM, IC, and PIs. Given the planned 18 months of data collection we anticipate 3-4 meetings. At the minimum we anticipate the IMM

This work is licensed under a Creative Commons Attribution-NonCommercial-NoDerivatives 4.0 International License.

will meet at the following time points: Prior to data collection start, at 6-9 months, possibly at 12 months, and at study completion (90 days after last patient enrollment).

The following data will be reviewed at the scheduled assessments (with the exception of the pre-data collection meeting): Subject data, Efficacy and Safety Outcomes, Enrollment, Assessment of minority recruitment, protocol violations/deviations, adverse events, study termination.

Adverse events attributable to the intervention or the performance of guideline concordant BPPV care processes are anticipated to be rare.

12 PUBLICATION OF RESEARCH FINDINGS

Publication of the results of this trial will be governed by the policies and procedures developed by the Executive Committee.

13 REFERENCES

1. von Brevern M, Radtke A, Lezius F, et al. Epidemiology of benign paroxysmal positional vertigo: a population based study. J Neurol Neurosurg Psychiatry 2007;78:710-5.
2. Lopez-Escamez JA, Gamiz MJ, Fernandez-Perez A, Gomez-Finana M. Long-term outcome and health-related quality of life in benign paroxysmal positional vertigo. Eur Arch Otorhinolaryngol 2005;262:507-11.
3. Fife TD, Iverson DJ, Lempert T, et al. Practice parameter: therapies for benign paroxysmal positional vertigo (an evidence-based review): report of the Quality Standards Subcommittee of the American Academy of Neurology. Neurology 2008;70:2067-74.
4. Bhattacharyya N, Baugh RF, Orvidas L, et al. Clinical practice guideline: benign paroxysmal positional vertigo. Otolaryngol Head Neck Surg 2008;139:S47-81.
5. Chang AK, Schoeman G, Hill M. A randomized clinical trial to assess the efficacy of the Epley maneuver in the treatment of acute benign positional vertigo. Acad Emerg Med 2004;11:918-24.
6. Brown MD. Evidence-based emergency medicine. Is the canalith repositioning maneuver effective in the acute management of benign positional vertigo? Ann Emerg Med 2011;58:286-7.
7. Hilton M, Pinder D. The Epley (canalith repositioning) manoeuvre for benign paroxysmal positional vertigo. Cochrane Database Syst Rev 2004:CD003162.
8. Munoz JE, Miklea JT, Howard M, Springate R, Kaczorowski J. Canalith repositioning maneuver for benign paroxysmal positional vertigo: randomized controlled trial in family practice. Can Fam Physician 2007;53:1049-53, 8.
9. Yimtae K, Srirompotong S, Sae-Seaw P. A randomized trial of the canalith repositioning procedure. Laryngoscope 2003;113:828-32.
10. Froehling DA, Bowen JM, Mohr DN, et al. The canalith repositioning procedure for the treatment of benign paroxysmal positional vertigo: a randomized controlled trial. Mayo Clin Proc 2000;75:695-700.

This work is licensed under a Creative Commons Attribution-NonCommercial-NoDerivatives 4.0 International License.

11. Lynn S, Pool A, Rose D, Brey R, Suman V. Randomized trial of the canalith repositioning procedure. Otolaryngol Head Neck Surg 1995;113:712-20.
12. Helminski JO, Zee DS, Janssen I, Hain TC. Effectiveness of particle repositioning maneuvers in the treatment of benign paroxysmal positional vertigo: a systematic review. Phys Ther 2010;90:663-78.
13. Polensek SH, Tusa R. Unnecessary diagnostic tests often obtained for benign paroxysmal positional vertigo. Med Sci Monit 2009;15:MT89-94.
14. Newman-Toker DE, Stanton VA, Hsieh YH, Rothman RE. Frontline providers harbor misconceptions about the bedside evaluation of dizzy patients. Acta Otolaryngol 2008;128:601-4.
15. Stanton VA, Hsieh YH, Camargo CA, Jr., et al. Overreliance on symptom quality in diagnosing dizziness: results of a multicenter survey of emergency physicians. Mayo Clin Proc 2007;82:1319-28.
16. Polensek SH, Tusa RJ, Sterk CE. The challenges of managing vestibular disorders: a qualitative study of clinicians' experiences associated with low referral rates for vestibular rehabilitation. Int J Clin Pract 2009;63:1604-12.
17. Kerber KA, Morgenstern LB, Meurer WJ, et al. Nystagmus assessments documented by emergency physicians in acute dizziness presentations: a target for decision support? Acad Emerg Med 2011;18:619-26.
18. Kerber KA, Burke JF, Skolarus LE, et al. Use of BPPV Processes in Emergency Department Dizziness Presentations: A Population-Based Study. Otolaryngology -- Head and Neck Surgery 2013;148:425-30.
19. Kerber KA, Meurer WJ, West BT, Fendrick AM. Dizziness presentations in U.S. emergency departments, 1995-2004. Acad Emerg Med 2008;15:744-50.
20. Kerber KA, Schweigler L, West BT, Fendrick AM, Morgenstern LB. Value of computed tomography scans in ED dizziness visits: analysis from a nationally representative sample. Am J Emerg Med 2010;28:1030-6.
21. Newman-Toker DE, Camargo CA, Jr., Hsieh YH, Pelletier AJ, Edlow JA. Disconnect between charted vestibular diagnoses and emergency department management decisions: a cross-sectional analysis from a nationally representative sample. Acad Emerg Med 2009;16:970-7.
22. Eagles D, Stiell IG, Clement CM, et al. International survey of emergency physicians' priorities for clinical decision rules. Acad Emerg Med 2008;15:177-82.
23. Woodworth BA, Gillespie MB, Lambert PR. The canalith repositioning procedure for benign positional vertigo: a meta-analysis. Laryngoscope 2004;114:1143-6.
24. Lopez-Escamez J, Gonzalez-Sanchez M, Salinero J. [Meta-analysis of the treatment of benign paroxysmal positional vertigo by Epley and Semont maneuvers]. Acta Otorrinolaringol Esp 1999;50:366-70.
25. von Brevern M, Seelig T, Radtke A, Tiel-Wilck K, Neuhauser H, Lempert T. Short-term efficacy of Epley's manoeuvre: a double-blind randomised trial. J Neurol Neurosurg Psychiatry 2006;77:980-2.
26. Cohen HS, Kimball KT. Effectiveness of treatments for benign paroxysmal positional vertigo of the posterior canal. Otol Neurotol 2005;26:1034-40.

This work is licensed under a Creative Commons Attribution-NonCommercial-NoDerivatives 4.0 International License.

Appendix A – Consent / Survey Script

Part 1a: No answer, leave message.
Hello, my name is … and we are doing a research study evaluating dizziness care in the emergency department. The National Institutes of Health funds this research. We are working with the emergency medicine doctors at area hospitals and that is how we learned about your recent visit. We have a brief survey for you about your care. If you are interested, it takes only about 5 minutes, and you can call me back at this number X. I will also try to call you back at another time. If you would prefer to complete an electronic version of the survey, you can text XXX-XXX-XXXX to start the text message version of the survey, or you can go to [internet address URL] to complete an online survey. Standard text messaging rates apply for the text message survey. Thank you. Bye.

Part 1b. Person answers.
Hello, my name is … and we are doing a research study evaluating dizziness care in the emergency department. The National Institutes of Health funds this research. We are working with the emergency medicine doctors at area hospitals and that is how we learned about your recent visit. We have a brief survey for you about your care. This is not related to your hospital stay, and you will not be penalized in any way if you do not participate or lose any benefits you normally would be entitled to.
This take only about 5 minutes. Are you willing to let me tell you more about it?

[Person says “no”]
OK, I understand. Would you be willing to talk at another time? Thanks for your consideration. Bye.

[Person says “yes”]
Ok, great. The answers here will NOT be part of your medical record. This survey is voluntary and takes about one to three minutes. If you don’t want to do this survey now, we could call back at another time that is convenient for you. There is no direct benefit to you for doing this survey, but you will help us learn about how to take care of future patients with dizziness. Whenever we conduct research, we need to let you know of any risks involved with your participation. With this study, there is a minimal risk of loss of confidentiality. We do put security measures in place to protect your information you share with me today as well as your health records. These measures include password protected files and computer databases, storing information in locked offices, and only allowing authorized research team members to have access your information. However, no system is perfect.

Do you want to participate in the brief survey?
Do you have any additional questions?

Part 2: Survey

This work is licensed under a Creative Commons Attribution-NonCommercial-NoDerivatives 4.0 International License.

The care for dizziness in the emergency department may involve different tests and physical exam findings. Not all tests or physical exam findings are needed for each patient, so just because we are asking about something, doesn't mean that you should have had that sort of test or physical examination done.

While you were in the emergency department, do you remember whether a physician, physician's assistant, nurse practitioner or nurse did a physical exam?
Yes / No / Unsure

At any point during the ED visit, did one of the medical providers move you somewhat quickly from a sitting up position to a laying down position with your head tilted back?
Yes / No / Unsure

At any point during the ED visit, did one of the medical providers try to treat your dizziness by first having you lay down somewhat quickly with your head turned to one side, then have you roll over to one side until you were, or almost were, looking down at the ground, and then have you sit up?
Yes / No / Unsure

At the end of your visit, did your medical provider refer you to some other provider to be tested and or treated for a condition called Benign Paroxysmal Positional Vertigo (can also be called just Benign Positional Vertigo, BPPV, or BPV)?
Yes / No / Unsure

Has your dizziness, unsteadiness or vertigo gone away?
Yes / No / Unsure / I didn't have those symptoms during the recent emergency department visit

Thank you for your time. Do you have any other questions?

Electronic Survey Versions

Text/Question 1: Before the survey begins you will receive 3 text messages with the risks and benefits of participating. If you have any questions, please call XXX-XXX-XXXX.

Text/Question 2: This survey is voluntary and your answers will not be part of your medical record. There is no direct benefit to you but what we learn may help future patients.

Text/Question 3: There is a minimal risk of loss of confidentiality. Security measures such as password protected databases are in place to protect your information.

Text/Question 4: If you would like to participate, please reply to this message with "yes".

This work is licensed under a Creative Commons Attribution-NonCommercial-NoDerivatives 4.0 International License.

Text/Question 5: When at the emergency department, do you remember if a doctor or someone else examined you? (YES or NO or UNSURE)

Text/Question 6: During that visit, did someone move you quickly from sitting to laying with your head tilted back? (YES or NO or UNSURE)

Text/Question 7: During that visit, did someone have you roll over in the bed or stretcher to treat your dizziness? (YES or NO or UNSURE)

Text/Question 8: After the visit, were you referred to another doctor or clinic for benign paroxysmal vertigo (BPPV or BPV)? (YES or NO or UNSURE)

Text/Question 9: Has your dizziness, unsteadiness or vertigo gone away? (YES or NO or UNSURE or NEVER HAD THOSE)

This work is licensed under a Creative Commons Attribution-NonCommercial-NoDerivatives 4.0 International License.

Appendix B

Independent Medical Monitor Plan – Version 1.0 26 January 2016
Trial Title: Partnered Implementation of Evidence Based Best Care Practice for Benign Paroxysmal Positional Vertigo: A Stepped Wedge, Randomized Controlled Clinical Trial

Data and safety monitoring plan

The trial will utilize an independent medical monitor (IMM).
The target population of the intervention is physicians. The implementation strategy aims to increase the appropriate use by physicians of the guideline supported test and treatment of benign paroxysmal positional vertigo. In the education part of the intervention, we will explicitly describe potential risks and contraindications. Providers will then be able to use their own judgment in identifying the appropriate patients. The reason for having oversight from an IMM is the potential risk of performing of BPPV processes inappropriately or inappropriate selection of patients. An additional risk is that providers exposed to the intervention may change their practice regarding test ordering (e.g., less ordering of head CT scans) which could result in missing a diagnosis that may have been made otherwise.

The IMM will be a neurologist or emergency physician experienced in the acute care of patients with dizziness generally and BPPV in particular. Funds to support these individuals have been included in the study budget.

Adverse events attributable to the intervention or the performance of guideline concordant BPPV care processes are anticipated to be rare.

The responsibilities of the IMM include the following:

1. Review and approve the research protocol and plans for data and safety monitoring. The IMM, in collaboration with the study leadership, will establish specific guidelines for monitoring for safety. This will include listing events that should be reported immediately to the IMM and the format of reporting cumulative data at intervals.
2. Review interim analyses of outcome data for safety and efficacy to determine whether the project should continue as originally designed, be changed, or be terminated. The IMM will review project performance information such as patient recruitment and retention, clinical center and resource center performance, and proposals for ancillary studies. It will provide advice to the investigators and the NIDCD on these topics. The IMM will recommend whether and to whom outcome results should be released prior to the reporting of study results.
3. Review the primary project abstract(s) and manuscript(s) with regard to determining that the results are fairly presented and the conclusions appropriate.
4. Review published reports of related studies submitted to him/her by NIDCD or the study leadership/investigators to determine whether the monitored study needs to be changed or terminated.
5. Review proposed modifications to the study prior to their implementation (e.g., increasing target sample size, modifying outcomes, etc.).

This work is licensed under a Creative Commons Attribution-NonCommercial-NoDerivatives 4.0 International License.

6. As soon as possible but within 10 business days following each scheduled meeting, provide the study leadership and the NIDCD with DRAFT written recommendations along with justification related to continuing, changing, or terminating the project.
7. As soon as possible but within 10 business days following each meeting, provide the study leadership and the NIDCD with a statement, where appropriate, concerning the impact on the trial of individually observed or cumulative adverse events. The study leadership will provide this information to each clinical center director to be shared with their IRBs.
8. Agree to otherwise keep information about the project confidential. Other than the communications with NIDCD and the study team described herein, the IMM will agree to keep his/her knowledge regarding information about the project (including, but not limited to, the protocol, the intervention, and the results) confidential until the public release of information via meeting presentations, published manuscripts, or other media.

The responsibilities of the Study Team include the following:

1. Handle all logistics related to each meeting or conference call, such as booking a group of rooms at a local hotel, providing information to the IMM, and providing timely reimbursement to each member following each meeting.
2. Work with the IMM to determine what information should be made available for review at each meeting to assist the IMM in carrying out its primary charge related to patient protection oversight, study operation, and data integrity.
3. At least 14 days prior to each meeting, the PI will provide a report to the IMM on current status of the trial, adverse events, and problems encountered (see Meetings for more details).
4. The PI is responsible for assuring that adequate notes are taken during the open, closed, and wrap-up sessions of each meeting and during each conference call so that draft minutes from each meeting or conference call can be prepared by the PI within 10 business days following the meeting, for review by the IMM and the IC representative. This review should be completed within 5 business days from receipt of the draft minutes. After receiving approval and/or comments from the IMM and NIDCD representative, the final minutes should be prepared by the PI and distributed to all meeting participants. The final minutes of a meeting or conference call should be available no later than 20 business days following the end of the meeting or conference call. The minutes should include general highlights of the discussions, general recommendations, action items, suggested protocol/study changes, and the rationale for each. Confidential data is not to be included in the minutes. The date for the next scheduled meeting with the IMM should be specified at the end of the minutes.
5. The NIDCD representative to the IMM meetings is responsible for: ensuring adequate and direct communication between the study leadership and the IMM; providing general advice to the study leadership and IMM related to operational issues; and keeping the NIDCD leadership apprised of the status of the trial and reviewing the recommendations presented by the IMM so that informed decisions related to acceptance of recommendations and their potential impact can be assessed.

This work is licensed under a Creative Commons Attribution-NonCommercial-NoDerivatives 4.0 International License.

Meetings:
The frequency of IMM meetings will be determined by the IMM, IC, and PI. Given the planned 18 months of data collection we anticipate 3-4 meetings. At the minimum we anticipate the IMM will meet with the study team at the following time points: Prior to data collection start, at 6-9 months, possibly at 12 months, and at study completion (90 days after last patient enrollment).

Each meeting may be divided into three parts:
1. An open session at which members of the clinical trial team, representatives from the NIDCD, and others may be present, at the request of the IMM, to review the conduct of the project and to answer questions. The focus in the open session should be on accrual, protocol compliance, and general safety issues. Outcome results by treatment group will not be discussed during this session.

2. A second, closed session involving the IMM and the unblinded statistician. The outcome results and safety information, if any, will be presented to the IMM by treatment group by the unblinded statistician.

3. A final wrap-up session is held, at which the IMM presents and discusses his or her recommendations, along with the rationale/justification for each, with the study leadership and the NIDCD representative.

Monitoring subjects and identifying adverse events: The mechanism used for monitoring subjects and identifying adverse events will be approved/determined by the IMM. The mechanism will likely include self-reporting by providers, review of medical records, and surveillance for return visits. Providers will be encouraged to report any possible adverse events to the study team and simple mechanisms to do so will be defined. The study team will conduct monthly scheduled assessments of study recruitment, data integrity and quality, adverse events, withdrawals, and compliance with protocol plan. The following data will be reviewed at the scheduled assessments: Subject data, Study Outcomes, Enrollment, Assessment of minority recruitment, Protocol violations/deviations, and adverse events.

This work is licensed under a Creative Commons Attribution-NonCommercial-NoDerivatives 4.0 International License.

Appendix C

Preliminary wording – minor revisions may occur in finalized document.

Apply patient sticker here<br><br>Please enter account # if sticker is not available

Please complete both Parts 1 & 2 by circling the appropriate response (Yes or No) as applicable

Part 1. BPPV Items:

1) Spontaneous or gaze nystagmus is absent (video link)<br><br>AND<br><br>2) Triggered, transient nystagmus on Dix-Hallpike Test is present (video links)	**Yes***	**No**

*Consider Epley maneuver (video link).

If patient improves and there are no other deficits:
BPPV is likely and serious pathology is unlikely

Part 2. Safety Items

Indicate whether or not the following are present by circling yes or no:

This work is licensed under a Creative Commons Attribution-NonCommercial-NoDerivatives 4.0 International License.

New focal neurologic deficits on exam or by report, such as:<br>• Dysarthria<br>• Visual field defect<br>• Ptosis<br>• Coordination problem<br>• Sensory loss<br>• Weakness	**Yes**	**No**
Vertical nystagmus when sitting still and looking straight ahead or to the side	**Yes**	**No**
Direction-changing nystagmus (left-beat looking to left, and right-beat looking to right)	**Yes**	**No**
Newly required assistance from another person or device to safely walk 10 feet (3 meters)	**Yes**	**No**
**Two or more** of the following:<br>• Age >= 60<br>• Initial SBP >=140, or DBP >=90<br>• Diabetes	**Yes**	**No**

Please note: Safety items are for research purposes only. Decisions about further testing and disposition should be made using your clinical judgment.

This work is licensed under a Creative Commons Attribution-NonCommercial-NoDerivatives 4.0 International License.
