# Supplementary material for: Implementation of evidence-based practice for benign paroxysmal positional vertigo: DIZZTINCT– A study protocol for an exploratory stepped-wedge randomized trial
Source: Trials. 2018 Dec 22;19:697. doi: 10.1186/s13063-018-3099-0 (PMC6303863; doi:10.1186/s13063-018-3099-0)
Supplement: Supplementary file 3 — Preliminary statistical analysis plan. (PDF 214 kb) [file 13063_2018_3099_MOESM3_ESM.pdf]

Statistical Analysis Plan
Dizztinct Trial
Version 1.0 Finalized 20 February 2018

1. Statistical design

Overview and hypotheses

The primary objective is to evaluate, in a community emergency department (ED) setting, the effect of the multi-faceted, theory-based educational intervention and implementation strategy on the use of the Dix-Hallpike test and Canalith Repositioning Maneuver in ED dizziness visits. The secondary objectives are to evaluate variation in BPPV process utilization at the provider level, care processes (ED length of stay, neuroimaging, inpatient hospitalizations), and health care costs.

The primary hypothesis is that patients seen at hospitals that have received the intervention will have a higher likelihood of receiving a BPPV care process (i.e., DHT, CRM) compared to patients seen at hospitals that have not received the intervention. The primary endpoint is measured at the individual patient level, and is binary (yes/no) characterizing the presence of documentation of either the Dix-Hallpike test, the Epley Maneuver (or CRM) (since the first step of the CRM is the diagnostic maneuver).

The secondary objective evaluates the performance of any BBPV care process, including performance of the DHT, or CRM or a referral to a BPPV care provider. We hypothesize that patient visits occurring at hospitals that have received the intervention will have a significantly different rate of having any of the BPPV care process endpoints (documentation of DHT or CRM or outpatient referral for BPPV evaluation to a BPPV provider) associated with the ED care compared to patient visits occurring at hospitals that have not received the intervention. These secondary endpoints are binary measured on the patient visit level.

The main safety hypothesis is that there will be no difference in the rate of index visit and short-term (90 day) stroke within the dizziness population aged 45 and older, in patients seen in EDs that have received the intervention versus patients seen in EDs that have not received the intervention. Safety secondary endpoint is defined as 90-day cumulative incidence of stroke in patients aged 45 and above following initial ED discharge home visit for dizziness (a survival endpoint).

Power and sample size

The trial will start with an initial no intervention period of approximately 4 months followed by randomized staggered intervention with a new hospital entering approximately every 2 months, finalized by approximately 4 post-intervention months will result in the approximately balanced number of 867 visits occurring without intervention and 933 visits occurring under (post) intervention. This calculation assumes the average anticipated total patient visit rate of 100 patients per month. Based on our pilot studies and the literature we expect the DHT or CRM procedure to be done in 5% patients before the intervention. With the expected number of visits calculated above, we will be able to detect the increased DHT or CRM rate of 9% and above with 90% power by a two-sided test at the

significance level of 5%. We expect a much bigger difference of 5% vs. 15% pre- vs. post-intervention DHT/CRM rates, respectively. Under this expected difference, we will have the power exceeding 99%. In fact, an order of magnitude smaller visit rate of 10.4 patients per month would be sufficient for 90% power under the anticipated difference. The reserves of power will be used to provide more power to fine-tune the multivariate mixed regression models and associated secondary analyses.

Recruitment plans

Each month, approximately 100 patients with dizziness seek care at the six hospital associated-emergency departments in Nueces County, Texas. Over an 18-month period we anticipate a total sample size of approximately 1,800. Approximately half of the 1,800 patient visits will occur prior to the intervention in the overall study. As dizziness volume can vary widely both within and across emergency departments we have provided maximum sample sizes for each site that are 200% of our initial estimates. In protocol Version 1.2, we amended our expected and maximum sample sizes, as reflected in "revised expected" and "revise maximum" in below table.

Anticipated and Maximum Enrollment by Site and Type

Site	Expected Patients	Maximum Patients	Revised Expected	Revised Maximum
Spohn Shoreline	400	800	1800	2800
Spohn Memorial	400	800	800	2000
Spohn South	200	400	1400	2000
CCMC – Doctors	350	700	1000	1750
CCMC – Bay Area / Heart	300	600	1200	1500
CCMC - Northwest	150	300	600	750

2. Analysis Plan

2.1 Preliminary evaluation of data:

Descriptive statistics will be run on the key endpoint variables (primary, secondary and safety), and key explanatory variables (presence of intervention, provider site, type of BPPV care process):

- frequencies on the presence of documentation of the DHT/CRM outcome (i.e., performing the DHT/CRM or referral for the DHT/CRM) (primary endpoint)
- Kaplan-Meier estimates of ED length of stay, assess the proportional Hazards assumption using log-log survival plots by group (defined by the presence of intervention).
- Frequencies of the use of neuroimaging
- Frequencies of inpatient hospitalizations
- Cumulative incidence functions for the incidence of stroke (safety endpoint). Graphical assessment of the Proportional Intensity assumption.
- Frequencies of missing data for each variable
- Identify features of the data that stand out and re-examine subject-specific data, if needed, for the presence of errors

2.2. Description of study process

The recruitment dynamics will be continuously analyzed to make sure the rate of subjects' entering the study is within the anticipated range. We will use cumulative intensity estimates that characterize the intensity of the recruiment process.

Subjects distribution over the intervention groups will be tracked t avoid substantial imbalance

Follow-up time will be assessed using Kaplan-Meier estimates.

Count and frequency of final completion across the subject for each provider will be tracked.

Subjects' data will be continuously entering data tables for the analysis, and the rate of evaluable subjects will be tracked as the study progresses.

2.3. Description of participants

Demographic characteristics (age, sex) will be analyzed by provider and intervention status using cross-tables

Risk factors for stroke will be summarized using hystograms, frequencies and cross-tables

2.4. Primary analysis/results

Methods/Approach

Primary outcome: Presence of documentation of the DHT/CRM outcome.

Primary analysis:

The primary analysis will use binary logistic regression and will include covariates for hospital, month (to handle secular trends), and intervention:

First approach: Generalized Linear Model (GLM) with logistic link

1. (DHT/CRM_documentation) ~ InterventionPeriod + Month + HospitalID + RandomIntercept(Attending_ed_1) . We expect about 10% dizziness patients to be suitable for a DHT/CRM.

   Hypothesis testing: A two-sided likelihood ratio test for Intervention regression coefficient at the significance level of 5%.

Results will be reported as parameter estimates, 95% confidence intervals, and 2-sided p-values from the likelihood ratio and Wald tests.

Missing data

Two analyses with and without missing data will be performed.

Predictive matching multiple imputation will be the method for the analysis including missing data. If the fraction of missing data does not exceed 16%, one round of imputation will be used; if exceeds, full multiple imputation machinery will be involved.

Analysis without missing data will include a complete dataset achieved by listwise deletion of subject with missing data.

We do not anticipate more than 15% missing data.

While we do not anticipate the results of these analyses to differ, a discussion will be provided should any differences exhibit themselves.

2.5. Secondary analyses

The following secondary analyses will be presented:

(a) Other analysis approaches on the primary measurement A.

The primary analysis described above is based on multivariable models that adjust for potential confounders. Significance of those confounders will be assessed using variable selection procedures (best possible model search using BIC, backward variable selection using likelihood ratio test). Confounders not included in the best model will be deemed non-significant.

(b) Other analysis approaches on the primary measurement B.

Assess the interaction of InterventionPrd with month in the primary model. This will explore the hypothesis that the intervention has an increasing effect over the months after the intervention period.

(c) Other analysis approaches on the primary measurement C.

Assess the interaction of InterventionPrd with hospital in the primary model. This will explore the hypothesis that the intervention differs in its effect by hospital.

(d) Other analysis approaches on the primary measurement D.

Assess the change in results with a more nuanced hierarchical categorization of the primary provider. We will replace attending_ed_1 in the primary model with a variable that has a hierarchy as follows: resident_ed_1 > midlevel_ed_1 > attending_ed_1. This hierarchy is based on the theory of provider with most influence on the outcome. An ideal analysis of the issue of multiple providers would have multiple levels of the individual providers, but this structure would be very difficult to fit given our anticipated number of outcomes.

(e) Other analysis approaches on the primary measurement F.

Mediation analysis. We will enter variables that could mediate the effect of the intervention into the primary model. Mediators assessed will be variables reflecting implementation fidelity. These include attendance at a CME session, registration on website (or time of website), download of app (or time on app), participation in post-intervention survey or interview.

(f) Other outcomes (secondary outcomes):

Analysis of secondary outcomes will be similar to the analysis of the primary outcome, except that a model appropriate for the response type will be used.

Use of neuroimaging and inpatient hospitalizations will be analyzed using multivariate GLM with logistic link.

ED length of stay, time to re-hospitalization, follow-up time will be assessed using multivariate Proportional Hazards (Cox) models.

(g) We will analyze data for possible trend of increasing use of BPPV diagnosis as a result of the intervention

2.6. Adverse effects

Adverse events will be tabulated with each treatment group:

- Number of events by type of event
- Number of subjects by highest grade of event

Multivariate multinomial logistic model will be used to test if the rates of adverse events vary by Intervention, Provider, Calendar Time.

Safety endpoint will be analyzed using the Proportional Intensity model for Cumulative incidence functions, where incidence is defined for stroke. 95% confidence intervals and a test for the efect of the Intervention binary variable will be included in the report from this analysis.

2.7 Subgroup analyses

Subset analyses. The analyses listed under “Primary Analyses”, including evaluation of the proportion of DHT in each of the subsets, will be repeated for the following specific subsets of the data:

1) First reason for visit is dizziness
2) First reason for visit is dizziness or first diagnosis outcome listed by the physician is a dizziness diagnosis (any of dizziness NOS, vertigo NOS, vestibular diagnosis).
3) First diagnosis outcome listed by the physician is a dizziness diagnosis (any of dizziness NOS, vertigo NOS, vestibular diagnosis).

Assess the change in results with a more specific dizziness population. Our population is very broad and the DHT/CRM are likely only relevant in 10-20% of the population. To render the population more likely to be DHT/CRM relevant, the primary analysis will be repeated in the sample with dizziness (rfv1 value of 1,2,3,4,or 5) as the #1 Reason for the Visit. [we will also consider including population with a dizziness diagnosis of DizzyNOS/VertigNOS/VestibularDO].

3. Post-hoc (data driven) analyses:

Should any of the models in Section 2 show violations of the key model assumptions, model modifications will be undertaken until the model is not rejected by the data.

Transformation os the explanatory variables will be explored if residual plots indicate more complex than loglinear form of the effect. Generalized additive models based on splines will be used to suggest an appropriate parametric form of the effect.

With the logistic GLMs alternative link functions will also be considered.

Non-proportional survival and intensity models for times to event and rate endpoints (length of stay in ED, follow up time, re-hospitalizations, follow-up time, incidence of stroke) based on frailties (gamma and stable frailty distributions will be explored) will be explored in case model diagnostics reveal departures from proportionality of hazards and intensities.

4. Attachments

Attached are files with R code (without the dependence libraries) implementing some of the procedures in the above SAP, using initial fraction of the data.

```
################################################################################
#  Primary analysis code
#  March 2018
################################################################################

wd = 'C:\\Users\\tsodikov\\Google Drive\\collaboration\\kerber\\IMM 2018\\IMMMarch2018'
setwd(wd)

################################################################################
# Set up a link to the source code library
# used in this project
# Note: insert the specific directory where you put the library
################################################################################

source('C:\\Users\\tsodikov\\Google Drive\\Useful functions\\sourceuf.r')

################################################################################
# Link to standard R packages
################################################################################

library(Hmisc)
library(pastecs)
library(car)
library(lattice)
library(plyr)
library(reshape2)
# help(package='reshape2')
library(nlme)
library(ggplot2)
library(lme4)
library(GGally)
library(ordinal)
library(Epi)

################################################################################
# Link to Alex's library of useful functions
################################################################################

sourceuf('DataFunctions.r')
sourceuf('Descriptives.r')
sourceuf('Hypotheses.r')
sourceuf('Strings.r')
sourceuf('Files.r')
sourceuf('Models.r')
sourceuf('LikelihoodRatio.r')
sourceuf('Rsquared.r')
sourceuf('Longitudinal.r')
sourceuf('Corr.r')

################################################################################
# Set up input and output directories (create if needed)
################################################################################

resdir = CombinePath(wd,"Results")
# create directory if does not exist
CreateDir(resdir)
# Clean the results directory
RemoveFiles(resdir)

datadir = file.path(wd,'Data')

################################################################################
# Read in data, merge files, some common sense checks
################################################################################

d = read.csv(file.path(datadir,'DIZTINCTPerEncounter_DATA_2018-03-06_0927.csv'))
nd = names(d)
dpi = read.csv(file.path(datadir,'DizztinctBasicOutcomeMatchMarch2018.csv'))
dpi$StrokeLink = TRUE
ndpi = names(dpi)

d$patient_account_num = as.character(d$patient_account_num)
table(d$patient_account_num)
dpi$patient_account_num = as.character(dpi$patient_account_num)
table(dpi$patient_account_num)

# merge by patient_account_num, visit_arrival_date
# variavles to keep from dpi
keepvardpi = c('patient_account_num', 'visit_arrival_date',"NarrativeDizztinct","NarrativeBASIC",'StrokeLink')

dm = merge(d,dpi[,keepvardpi],by=c('patient_account_num','visit_arrival_date'),all=T)
dm$StrokeLink[is.na(dm$StrokeLink)] = FALSE
# View(d)
# View(dpi)
# View(dm)
```

```
missingMRNs = setdiff(d$patient_account_num,dm$patient_account_num)
# View(d)

nrow(d)

range(table(d$patient_account_num))
range(table(d$patient_mrn))
range(table(dm$patient_account_num))
length(table(dm$patient_account_num))
length(table(dm$patient_mrn))
range(table(dm$patient_mrn))

################################################################################
# Descriptives for the IMM 2018 report
################################################################################

# View(d)
FindNames(nd,'DHTorEpleyPerform')

table(dm$DHTorEpley,useNA='always')

mytabci(dm$StrokeLink)
nrow(dpi)

mytabci(dm$DHTorEpleyPerform)

mytabci(dm$DHTorEpleyPerform)

dm$postintervention
mytabci(dm$postintervention)

filter = dm$postintervention==0
mytabci(dm$DHTorEpleyPerform[filter])
filter = dm$postintervention==1
mytabci(dm$DHTorEpleyPerform[filter])

filter = dm$postintervention==0
mytabci(dm$StrokeLink[filter])
table(dm$DStrokeLink[filter])
filter = dm$postintervention==1
mytabci(dm$StrokeLink[filter])

mytable(dm$StrokeLink)
mytable(dm$postintervention)

table(dm$StrokeLink,dm$postintervention)

################################################################################
# Preliminary code for primary analysis
################################################################################

m = glmer(
          DHTorEpleyPerform~postintervention+(1|patient_mrn)
          # + Age + Gender
          # +(1|Provider)
          # + (1|hospital_id)
          ,data=dm
          ,family=binomial(link = "logit")
          )

# print the model results without correlations, fixed effects
print(m, corr = FALSE)

# more detailed results with CI and hypotheses tests for hte effects
sm  = summary(m)
sm

MainHypothesisPvalue = sm$coef['postintervention','Pr(>|z|)']
MainHypothesisPvalue
```
